# Supplementary material for: A pan-cancer screen identifies drug combination benefit in cancer cell lines at the individual and population level
Source: Cell Rep Med. 2024 Aug 20;5(8):101687. doi: 10.1016/j.xcrm.2024.101687 (PMC11384948; doi:10.1016/j.xcrm.2024.101687)
Supplement: Document S1. Figures S1–S6 [file mmc1.pdf]

Cell Reports Medicine, Volume *5*

Supplemental information

A pan-cancer screen identifies drug combination benefit in cancer cell lines at the individual and population level

Daniel J. Vis, Patricia Jaaks, Nanne Aben, Elizabeth A. Coker, Syd Barthorpe, Alexandra Beck, Caitlin Hall, James Hall, Howard Lightfoot, Ermira Lleshi, Tatiana Mironenko, Laura Richardson, Charlotte Tolley, Mathew J. Garnett, and Lodewyk F.A. Wessels

A pan-cancer screen identifies drug combination benefit in cancer cell lines at the individual and population level

Daniel J. Vis[1#], Patricia Jaaks[2#], Nanne Aben[1#], Elizabeth A. Coker[2], Syd Barthorpe[2], Alexandra Beck[2], Caitlin Hall[2], James Hall[2], Howard Lightfoot[2], Ermira Lleshi[2], Tatiana Mironenko[2], Laura Richardson[2], Charlotte Tolley[2], Mathew J. Garnett[2,*], Lodewyk F.A. Wessels[1,4,*]

[1] Division of Molecular Carcinogenesis, The Netherlands Cancer Institute and Oncode Institute, Amsterdam 1066 CX, The Netherlands
[2] Wellcome Sanger Institute, Cambridge, UK
[3] Department of EEMCS, Delft University of Technology, Delft 2628 CD, the Netherlands
[4] Cancer Genomics Netherlands, Uppsalalaan 8, Utrecht 3584CT, the Netherlands
[#] Equal contributors
* Corresponding authors

Supplemental Figures

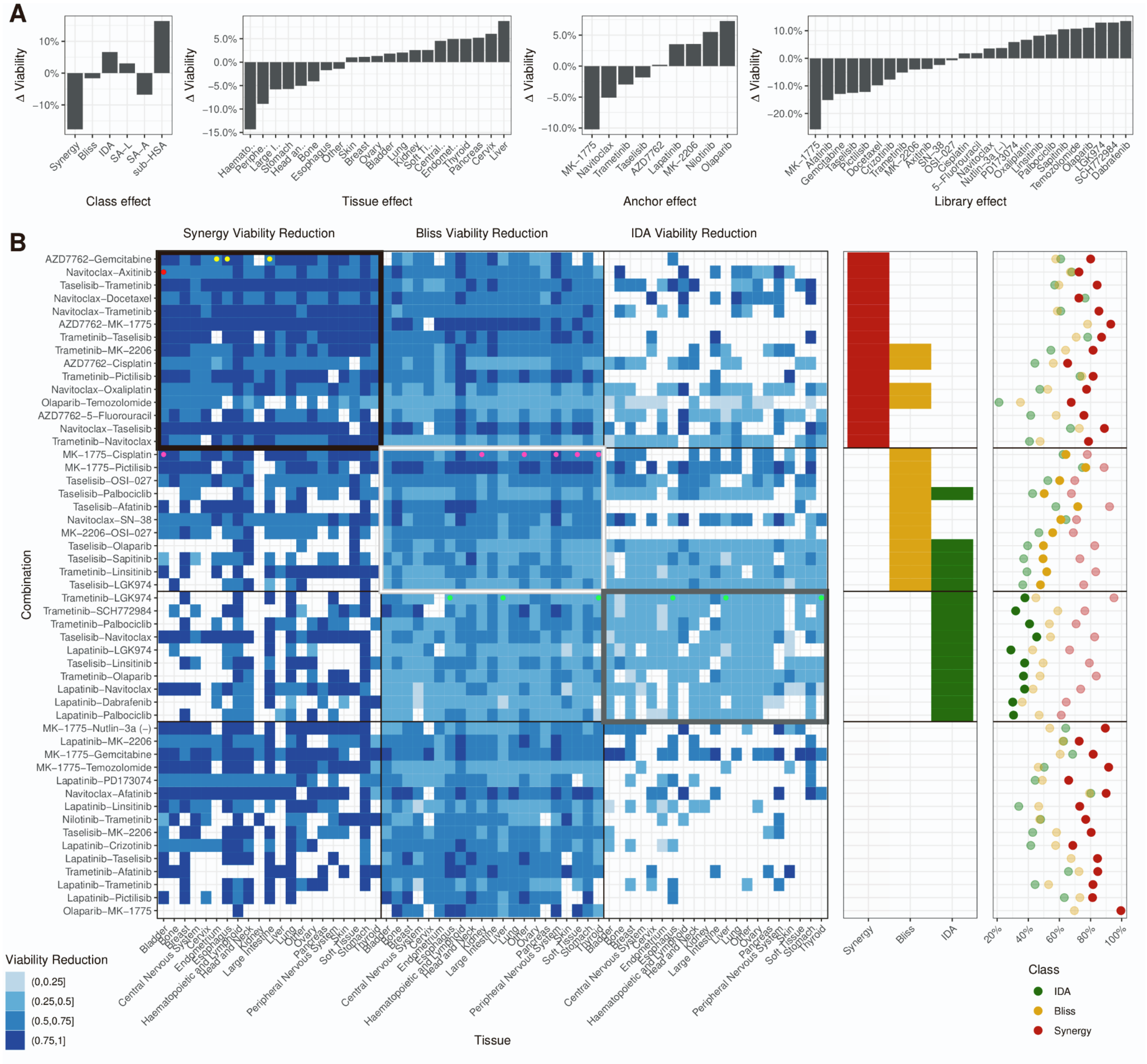

Supplemental Figure 1. **Outcome of classification of responses, related to Figure 3** (A) Decomposition of viability for individual response classes, tissue effects, anchor effects and library effects, show the general attribution of effects and their levels. (B) Mean viability reduction by tissue, combination, and combination benefit class. Empty cells mark zero class frequency. Marginal percentages are provided for combinations (right). Yellow dots indicate the drug pair-tissue combinations mentioned in the main text.

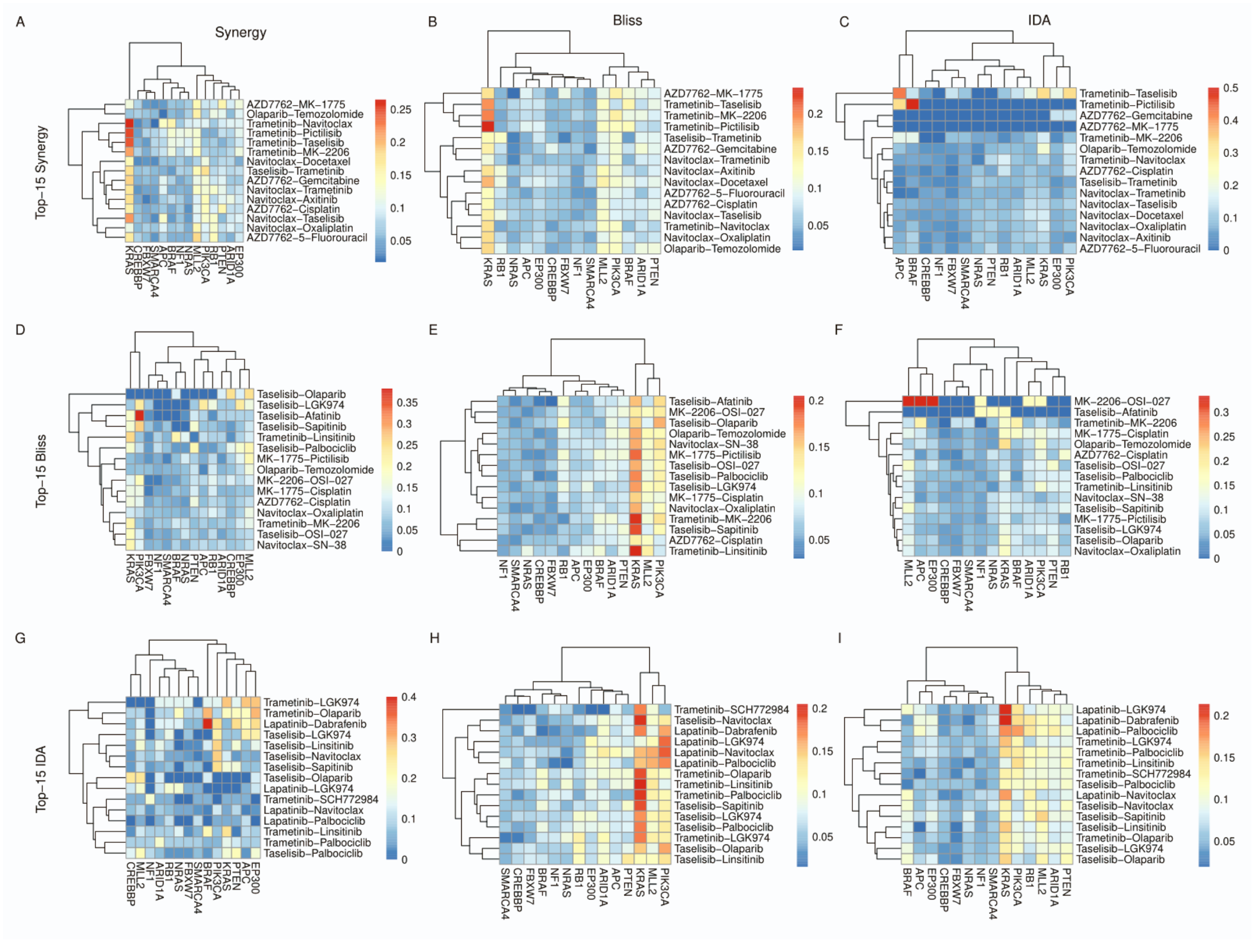

Supplemental Figure 2: **Clustered heatmaps for each of the response classes showing mutation frequencies as a function of the top 15 response classes, grouped by class, related to Figure 3**. The first row shows the top 15 synergy, the second top 15 Bliss, and the third top 15 IDA. The first column shows the mutation rates from the synergy class, the middle column from Bliss, and the third column from IDA. TP53 has been removed due to its high mutation rate to reveal more subtle patterns in the other genes. Legend indicates the mutation rate (0-1) for that class/combination.

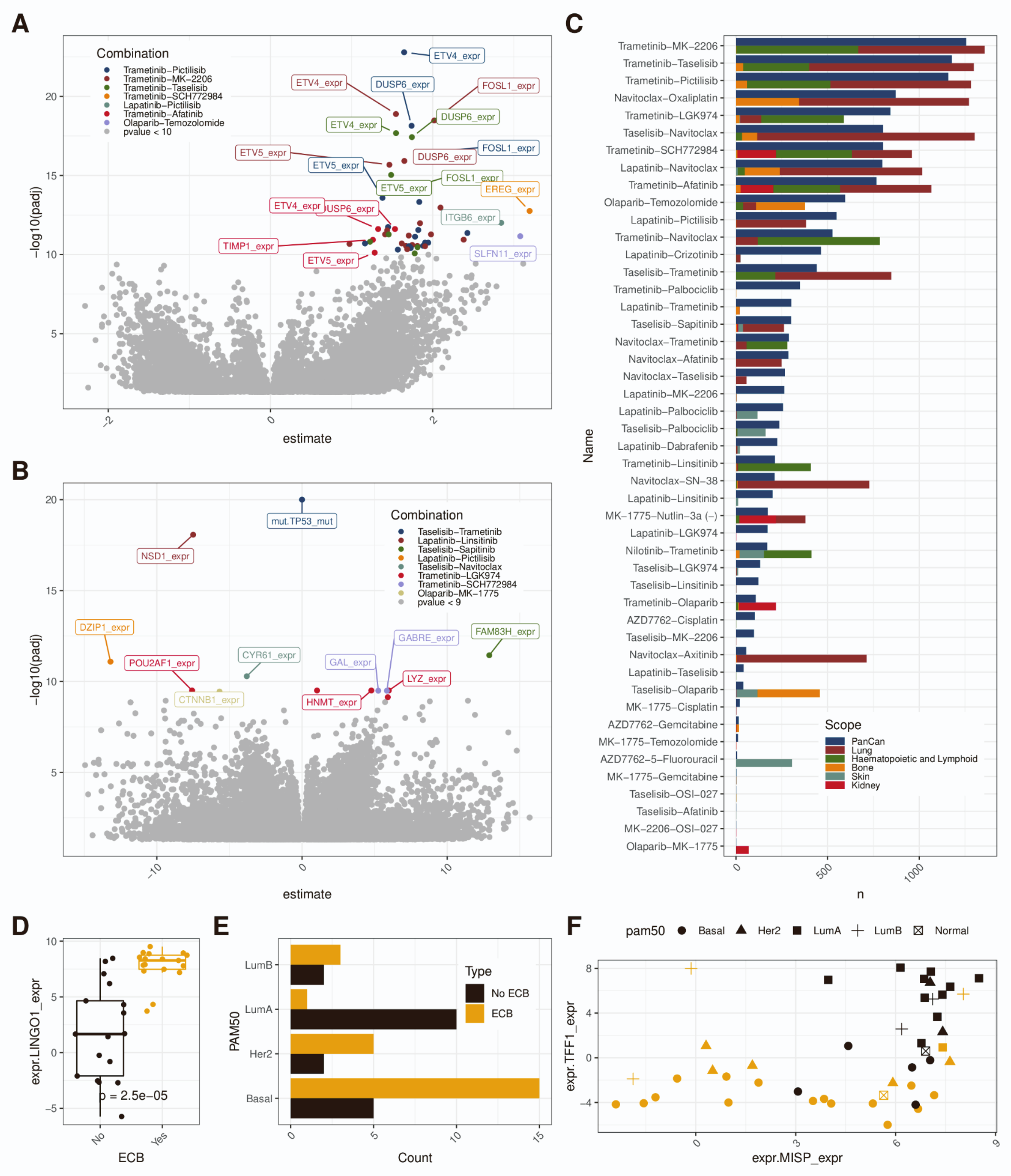

Supplemental Figure 3. Biomarkers for ECB status across and within tissues, related to Figure 4. (A) Volcano plot of pan-tissue biomarkers, showing effect size against p-value for the top five combinations per combination, labeling only the very strong associations. (B) Volcano plot of per-tissue biomarkers, -log10(p-adj) clipped at 20. (C) Count distribution of biomarkers within and across tissues. (D) Association of LINGO1 expression and ECB status in bone, boxplot shows median and 25% and 75% quantiles. (E) PAM50 subtypes and ECB status for MK1775+cisplatin

in breast. (G) Scatterplot displaying link between expression of TFF1 and MISP and PAM50 subtype.

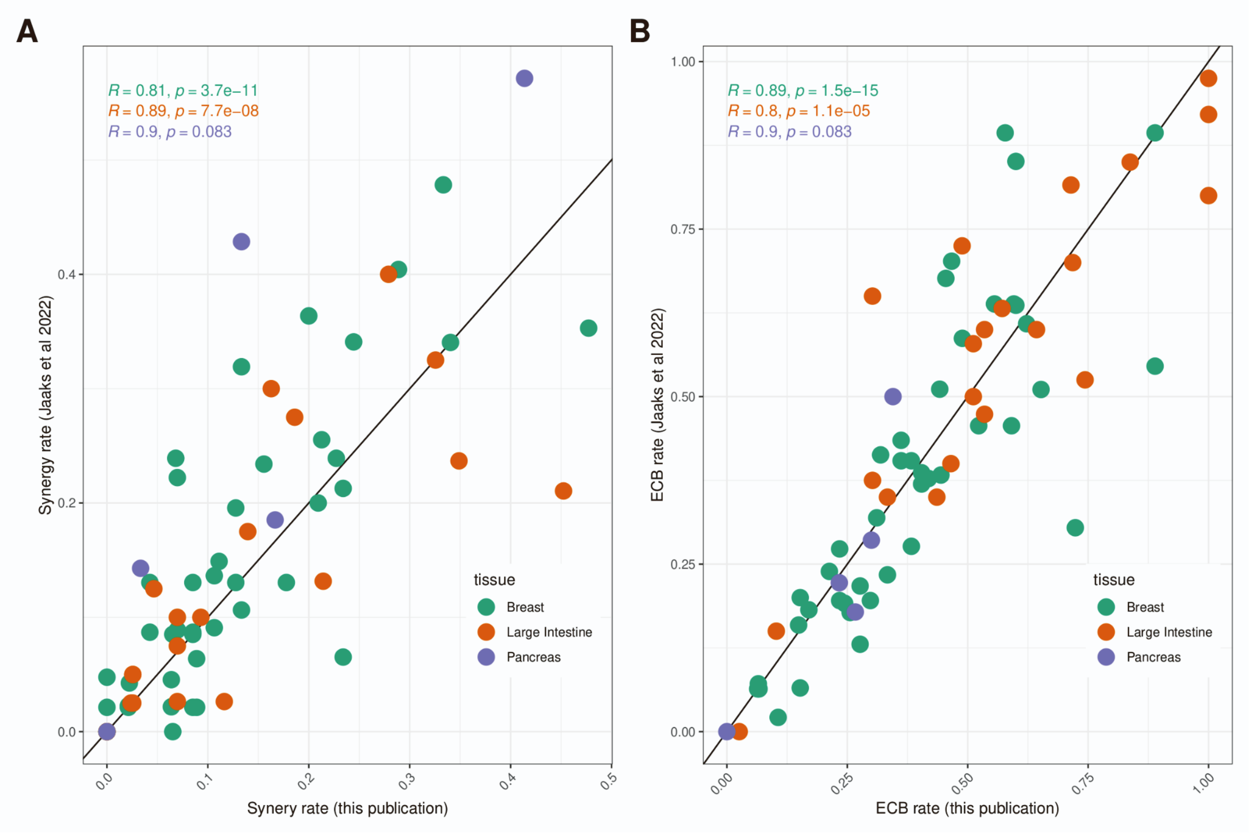

Supplemental Figure 4. Synergy and ECB rate comparison with independent screening data, related to Figure 4. (A) The synergy frequency for combinations with the same concentrations in both screens, by tissue type, and the associated per-tissue correlation. (B) The ECB frequency for the combinations with the same concentrations in both screens and their correlation by tissue type, and the associated per-tissue correlation.

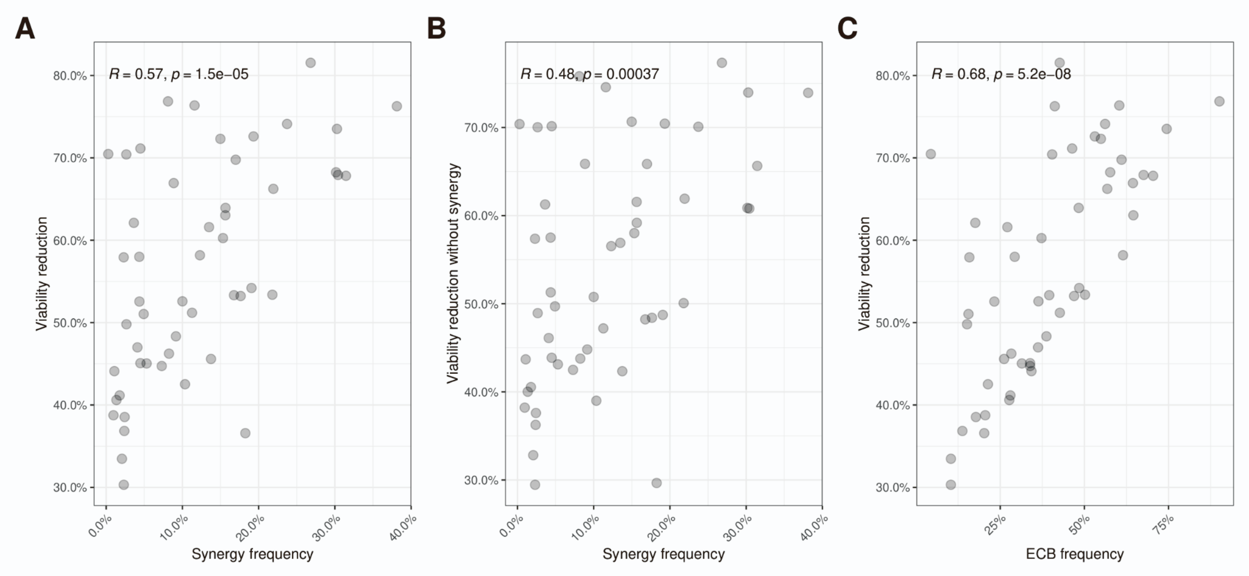

Supplemental Figure 5. Relation between synergy and efficacy, related to Figure 4. (A) Synergy count against mean viability reduction by combination. (B) Synergy count against mean viability reduction without synergy. (C) ECB count against mean viability reduction by combination.

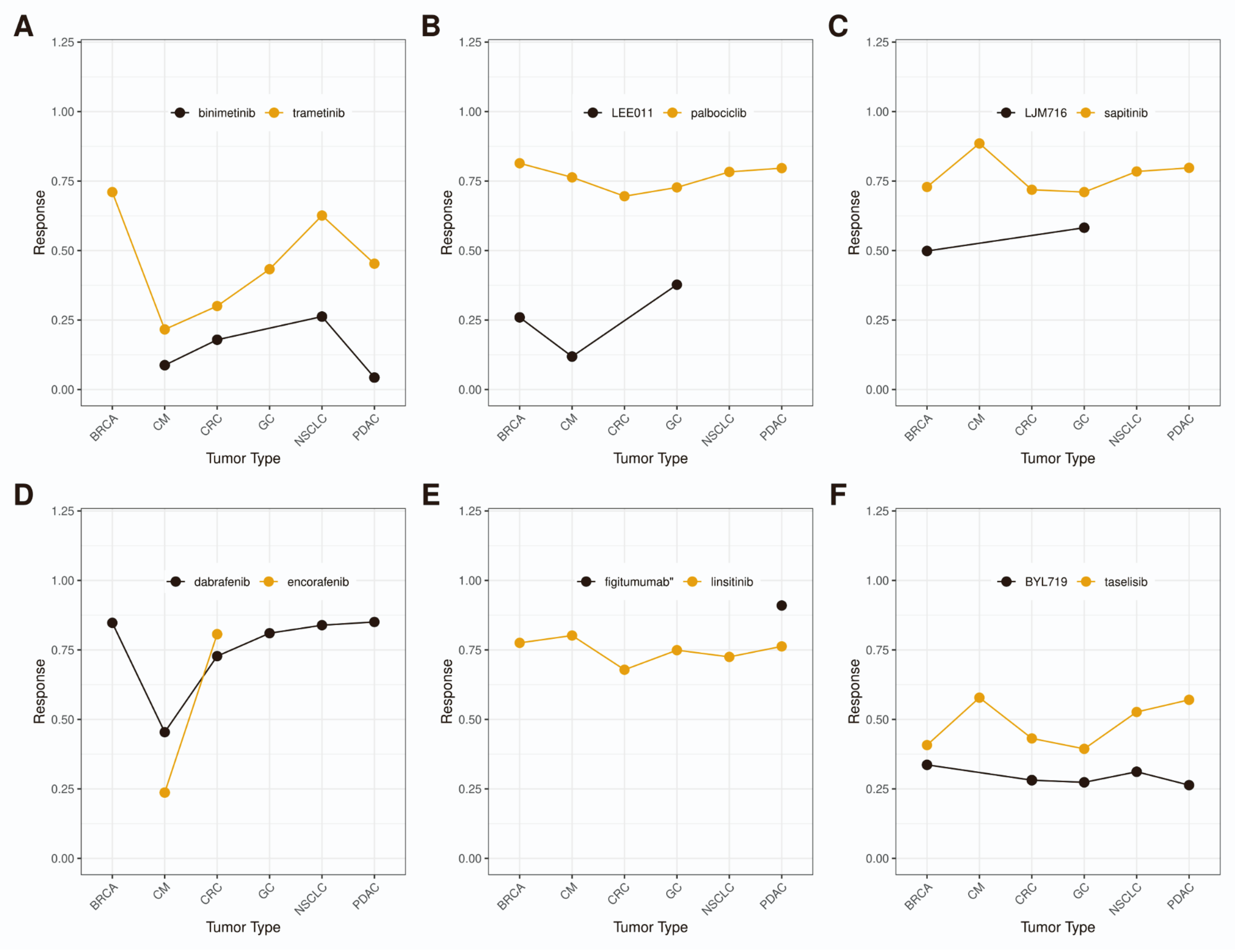

Supplemental Figure 6. Comparison of single agent effect for target-matched compounds in PDX and cell lines, related to Figure 5. (A) The hazard ratio for binimetinib (PDX, black) and mean Emax (viability at the highest concentration) for binimetinib (cell lines, yellow) show an effect of the single agent. (B) The hazard ratio for LEE011 (PDX, black) and mean Emax for palbociclib (cell lines, yellow) reveal an effect in PDX but resistance in cell lines. (C-F) Comparisons for other single-agent responses.

Supplemental Data S1: Source data and code (Data S1.zip) used for classification and figures generation, related to figures 1-5.
