# Supplementary material for: A pan-cancer screen identifies drug combination benefit in cancer cell lines at the individual and population level
Source: Cell Rep Med. 2024 Aug 20;5(8):101687. doi: 10.1016/j.xcrm.2024.101687 (PMC11384948; doi:10.1016/j.xcrm.2024.101687)
Supplement: Document S2. Article plus supplemental information [file mmc8.pdf]

Cell Reports
Medicine

Article

A pan-cancer screen identifies drug combination benefit in cancer cell lines at the individual and population level

Graphical abstract

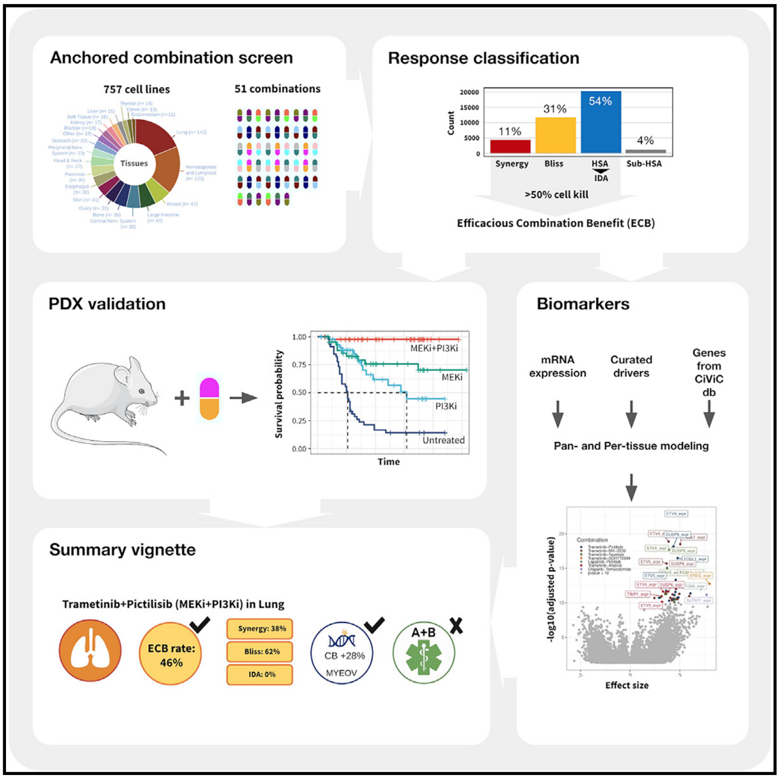

Authors

Daniel J. Vis, Patricia Jaaks, Nanne Aben, ..., Charlotte Tolley, Mathew J. Garnett, Lodewyk F.A. Wessels

Correspondence

mg12@sanger.ac.uk (M.J.G.),
l.wessels@nki.nl (L.F.A.W.)

In brief

Vis et al. developed an approach to model combination drug effects, identifying efficacious combination benefit (ECB) responses driven by synergy, Bliss, and independent drug action. They identify biomarkers of ECB and show that ECB is better than synergy at predicting patient response. A valuable resource for preclinical evaluation is provided.

Highlights

- Efficacious combination benefit (ECB) combines effective synergy, Bliss, and IDA responses
- ECB identifies a broad set of combination treatments with potential clinical benefits
- ECB predicts PDX response better than synergy alone
- Provides framework and large-scale data (757 cell lines) for preclinical development

Vis et al., 2024, Cell Reports Medicine *5*, 101687
August 20, 2024 © 2024 The Authors. Published by Elsevier Inc.
https://doi.org/10.1016/j.xcrm.2024.101687

CellPress

Cell Reports Medicine

CellPress
OPEN ACCESS

Article

A pan-cancer screen identifies drug combination benefit in cancer cell lines at the individual and population level

Daniel J. Vis,[3,5] Patricia Jaaks,[2,5] Nanne Aben,[1,5] Elizabeth A. Coker,[2] Syd Barthorpe,[2] Alexandra Beck,[2] Caitlin Hall,[2] James Hall,[2] Howard Lightfoot,[2] Ermira Lleshi,[2] Tatiana Mironenko,[2] Laura Richardson,[2] Charlotte Tolley,[2] Mathew J. Garnett,[2,*] and Lodewyk F.A. Wessels[1,4,6,*]
[1]Division of Molecular Carcinogenesis, Netherlands Cancer Institute, Amsterdam, the Netherlands
[2]Wellcome Sanger Institute, Cambridge, UK
[3]Department of EEMCS, Delft University of Technology, the Netherlands
[4]Oncode Institute, Utrecht, the Netherlands
[5]These authors contributed equally
[6]Lead contact
*Correspondence: mg12@sanger.ac.uk (M.J.G.), l.wessels@nki.nl (L.F.A.W.)
https://doi.org/10.1016/j.xcrm.2024.101687

SUMMARY

Combining drugs can enhance their clinical efficacy, but the number of possible combinations and inter-tumor heterogeneity make identifying effective combinations challenging, while existing approaches often overlook clinically relevant activity. We screen one of the largest cell line panels (N = 757) with 51 clinically relevant combinations and identify responses at the level of individual cell lines and tissue populations. We establish three response classes to model cellular effects beyond monotherapy: synergy, Bliss additivity, and independent drug action (IDA). Synergy is rare (11% of responses) and frequently efficacious (>50% viability reduction), whereas Bliss and IDA are more frequent but less frequently efficacious. We introduce "efficacious combination benefit" (ECB) to describe high-efficacy responses classified as either synergy, Bliss, or IDA. We identify ECB biomarkers *in vitro* and show that ECB predicts response in patient-derived xenografts better than synergy alone. Our work here provides a valuable resource and framework for preclinical evaluation and the development of combination treatments.

INTRODUCTION

Drugs used in combination can potentiate treatment efficacy. In a meta-analysis of 95 randomized anticancer clinical trials of non-cytotoxic agents, combination treatments had superior efficacy and prolonged overall patient survival over single agents.[1]

Combinations of chemotherapeutics have been empirically developed and refined in patients, typically over decades, and remain standard-of-care treatments.[2] Possible drug combinations rapidly increase as more targeted molecules enter clinical use, rendering efficient (clinical) exploration of the combination treatment space intractable.

Preclinical approaches can help to identify and prioritize new candidate combinations and the context in which they are active. Specifically, panels of molecularly annotated cancer cell lines have been used for pharmacogenomic profiling of small-molecule inhibitors and linking sensitivity with the genetic and molecular features of cells.[3–5] Several studies have explored parts of the combination space,[6–10] but they prioritized the number of combinations over the number of models screened. For example, the NCI Almanac used the NCI-60 pan-cancer panel to screen over 5,000 pairs of Food and Drug Administration (FDA)-approved cancer drugs but included few cell lines per tissue (for instance, skin and lung were only represented by nine cell lines each). "GDSC combinations" screened 2,000 drug combinations in 125 cell lines, but only from 3 cancer types.[10] The limited number of models and cancer types constrain in-depth, tissue-specific investigations such as biomarker detection or achieving a better understanding of the mechanisms underlying combination treatments.

Studies on combination effects generally contrast synergy with additivity,[7,8] despite additive effects inducing clinically meaningful patient responses. Furthermore, focusing on drug interactions within a cell line does not capture potential combination treatment benefits observable across a treated population of patients, a concept referred to as independent drug action (IDA)[11] that has been applied to the clinical setting.[12]

Here, we evaluated sensitivity to 51 clinically relevant combinations across 757 cancer cell lines, covering most cancers by tissue type, allowing the investigation of drug combination responses within and across heterogeneous cancer cells. We considered potency (reduction in IC50) and efficacy (reduction in viability at maximal drug concentration) and linked activity with genomic features. Notably, we departed from a synergy-exclusive approach

 © 2024 The Authors. Published by Elsevier Inc.
This is an open access article under the CC BY license (http://creativecommons.org/licenses/by/4.0/).

to introduce the concept of efficacious combination benefit (ECB), which describes active candidate drug combinations that are efficacious (>50% viability reduction) at the level of individual cell lines (either through synergy or Bliss additivity) and across populations of cancer cells (IDA).

The drug combination screen

We measured the effect on cell viability of 51 two-drug combinations in 757 cancer cell lines, generating dose-response quantifications of 37,900 unique combination-cell line pairs derived from 4.5 million measurements.

The cell lines included 21 tissues (Figure 1A and Table S1A), representing the tissue types of 92.6% of all estimated new cancer cases for 2021 (USA, ACS 2021). The number of cell lines per tissue type is representative of the relative annual incidence of cases in the United States (r = 0.6, *p* value = 0.01) (Table S1B).

We selected combinations of drugs used as monotherapies in the clinic that target essential proteins and pathways implicated in cancer and therapy response, drug combinations with the biggest potential based on literature review, or target combinations based on a mechanistic understanding. The screened drugs contained targeted compounds (*N* = 22) and chemotherapeutics (*N* = 7), and 16 of 29 are approved to treat cancer (Figure 1B; Tables S1C and S1D), underscoring the translational relevance of this screen.

We used an established pipeline for screening combinations and modeling responses.[10] Specifically, we screened anchor drugs at two concentrations in combination with library drugs screened over a 1,000-fold concentration range at seven concentrations (Figure 1C). This fits our signal-finding objective and is 71% more efficient than a full matrix design. We used a non-linear mixed effect model for curve fitting[10,13] and defined the IC50 as the concentration at which the combination reduces viability by 50%. At the same time, Emax represents the observed combination viability at the maximum concentration screened. We combined the anchor and library drug responses to obtain the expected combination response based on the Bliss independence model[14] (Figure 1D). We characterized drug interaction effects based on shifts in potency (ΔIC50, circles) and efficacy (ΔEmax, squares) that occur between the observed response ("Combination" in Figure 1D) and the Bliss expectation ("Bliss" in Figure 1D). While ΔIC50 and ΔEmax correlate (r = 0.84), each captures a unique population of combination responses (Figure 1E). Finally, combinations that reduce viability by 50% or more at the highest test concentration were defined as efficacious responses.

A classification system of drug combination response

We use a hypothetical dataset to illustrate our multi-step classification of cell line drug combination response based on synergy, Bliss, highest single agent (HSA), and IDA (Figure 2). We start by treating 11 colorectal (C) and nine lung (L) cell lines (Figure 2A) with a combination (Figures 2B and 2C). We obtain a set of drug response parameters for each cell line from the screening data and use them for response classification (Figure 2D). Specifically, in addition to (combination) Emax, ΔIC50, and ΔEmax, we quantified the Bliss viability at the highest screened library concentration (Bliss_Emax) and the HSA response at the highest screened library concentration (HSA_Emax) (Figures 1D and 2D).

For classification, we initially focus on individual cell lines (Figure 2E). A cell line response is synergistic when the observed combination is at least 8-fold more potent than Bliss expectation (Figure 2E #1). In our example, only C1 and L1 are classified as synergistic based on potency. Alternatively, based on efficacy, we classify a cell line response as synergistic when Emax is at least 20% lower than Bliss_Emax (Figure 2E #2). C2 and L1 satisfy this rule. The remaining cell lines are classified as Bliss when Combi_Emax is closer to Bliss_Emax than to HSA_Emax, provided that Bliss_Emax differs from HSA_Emax by a margin larger than the observed measurement noise of 7% viability (95% CI) and ΔIC50 and ΔEmax are positive (Figure 2E #3). Cell lines are classified as sub-HSA when Combi_Emax is higher than HSA_Emax by a margin exceeding the 7% noise level (Figure 2E #4). All remaining cell lines are classified as HSA. We apply these rules at both anchor concentration levels and retain the most favorable classification for each cell line.

Next, we consider combination responses at the population level instead of combination responses of individual cell lines (Figure 2F). This identifies combinations where the benefit over single drugs only becomes apparent at the population level, a concept termed IDA.[11] Since beneficial combination responses (synergy and Bliss) or lack thereof (sub-HSA) at the single-cell line level have already been identified, the population level classification is only applied to cell lines assigned to the HSA class for a given combination. We define a population as all cell lines with the same tissue of origin. For the two tissues in this example (colon and lung), we first identify the instances where at least 90% of the HSA-classified responses are driven by the library drug alone (Figure 2F #1). When this is the case for a given tissue of origin, we classify all the HSA cell lines in that tissue as "single-agent library". Similarly, if >90% of the HSA responses are driven by the anchor drug alone, all lines in that tissue are classified as "single-agent anchor" (Figure 2F #2). The remaining cases are where the response in a population is driven by the independent activity of the two drugs and are therefore classified as IDA.

The frequency and viability effects of population response classes

We classified all responses following the schema in Figure 2 and found, across the 37,900 combination-cell line pairs, that synergy and sub-HSA were comparatively rare (12% and 4%, respectively), with the majority of pairs assigned to Bliss (31%) or HSA (54%) (Figure 3A). Notably, 80% (41 out of 51) of combinations showed benefit over a single agent in more than 200 cell lines, resulting in classification as either synergy or Bliss.

We observed IDA frequently, representing 45% of HSA combination-tissue pairs (Figure 3B). The median IDA rate across tissue-drug combinations was 28% (interquartile range [IQR]: 8%–44%), with a median Emax of 55% (IQR: 38%–62%). We found that 35% of IDA-classified cell line responses were efficacious (Emax <50%). We used a mixed model to summarize the data and attribute efficacy changes to response class, tissue, and drug combination. We found that hematopoietic and lymphoid lines were the most sensitive and that liver was the most resistant tissue lineage, consistent with previous observations about single-agent sensitivity[3] (Figure S1A).

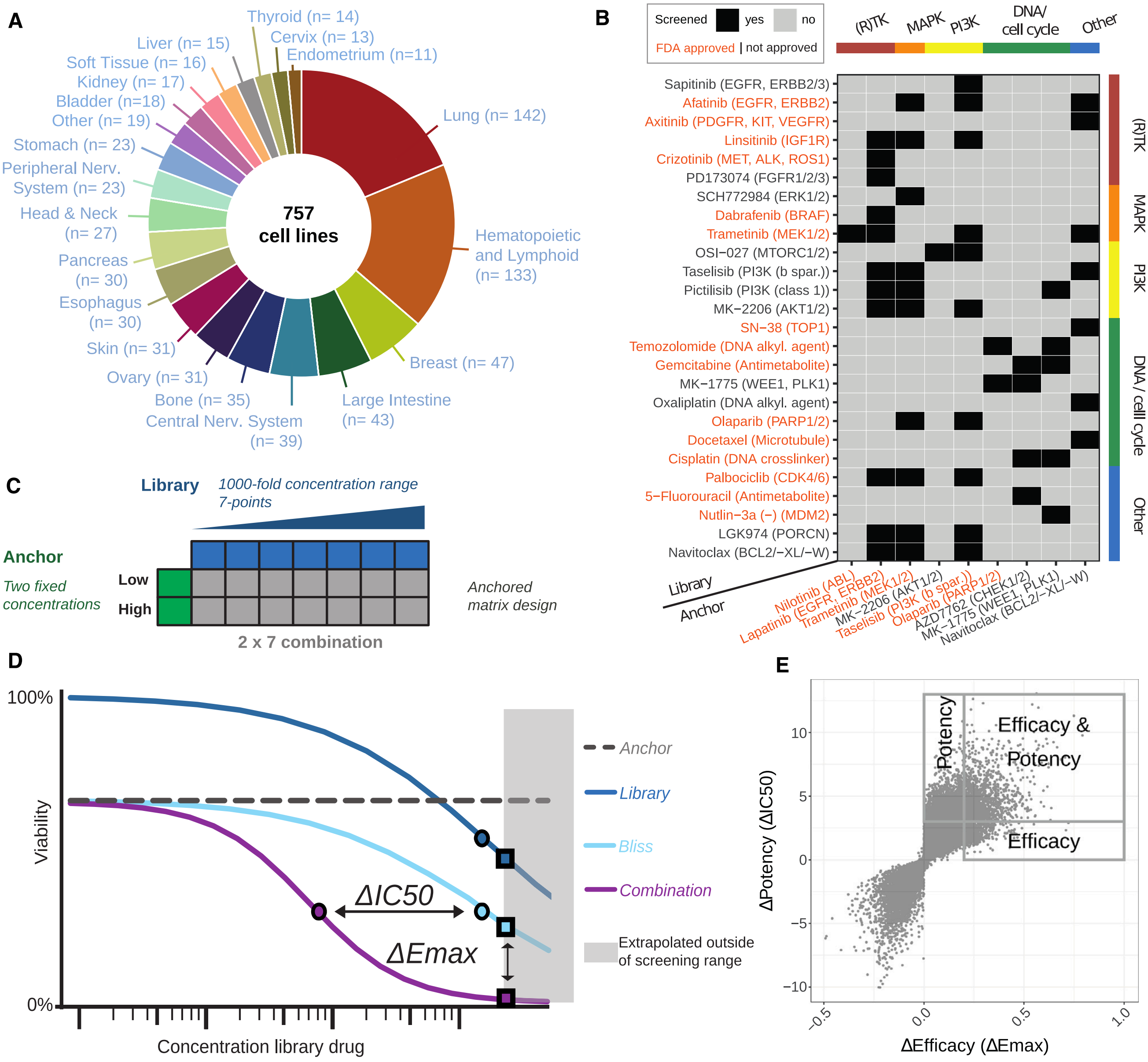

Figure 1. Study design and analysis approach

(A) Cell line panel from 21 cancer tissues.

(B) Table depicting the drug combinations screened with drug names, drug targets (in brackets), and their target pathways represented in rows and columns. Black squares mark screened combinations.

(C) The anchored matrix screening design.

(D) We quantified drug combination effects based on shifts in IC50 (ΔIC50) and Emax (ΔEmax) obtained by comparing the observed ("Combination") to the expected ("Bliss") response, with the IC50 and Emax values indicated by the circles and squares, respectively. We computed the Bliss response from the single drug anchor ("Anchor") and library ("Library") responses. The highest single agent (HSA) is the compound where library Emax or anchor viability is the lowest.

(E) Scatterplot of the 37,900 ΔPotency and ΔEfficacy data points from the screen.

Next, we focused on the top 15 synergy, Bliss, and IDA combinations, and we called these sets *groups*. These groups partially overlap and we used indicator variables to show this (Figure 3C, middle panel). Within each group, drug combinations were ranked based on the frequency of occurrence of the associated class. Together, these three groups comprised 36 of the 51 combinations (Figures 3C and 1B). We ranked the remaining combinations on the frequency of synergy.

The synergy group had a median tissue synergy rate of 23% and a median Bliss rate of 35%. The combination most frequently showing synergy was AZD7762 + gemcitabine (CHEK1/2, DDA), for which we found synergy in more than

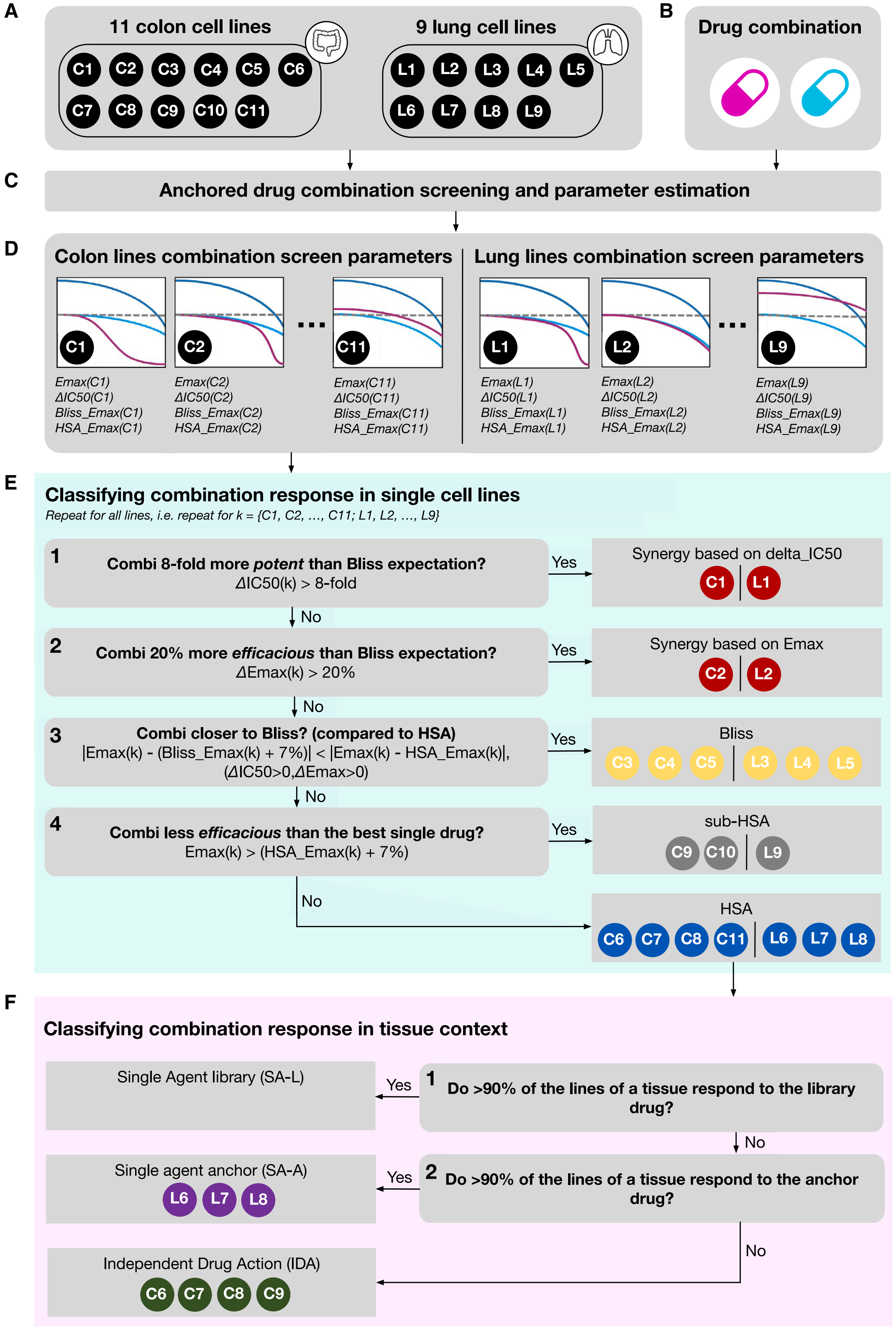

(legend on next page)

50% of the cell lines in the large intestine, esophagus, and endometrium (Figure 3D, yellow dots). The Bliss responses for this combination were efficacious in 68% of the tissues (13/19) (Emax <50%) (Figure S1A). Another example with high synergy rates (median = 33%) is navitoclax + axitinib (BCL2/XL/W, VEGFR1-3/PDGFR/c-KIT) and relatively high Bliss rates (median = 37%) across tissues, with 82% (14/17) of bladder cell lines showing synergy (Figure 3C, red dot).

The Bliss group combinations (five combinations overlapped with the *synergy group*, see indicator variables) had median Bliss rates of 47% and median synergy rates of 5%. The top *Bliss group* combination was MK-1775 + cisplatin (WEE1/PLK1, DNA crosslinking agent) and had Bliss rates exceeding 50% for most tissues, with five tissues (soft tissue, thyroid, kidney, peripheral nervous system [PNS], and other) showing rates exceeding 75% (Figure 3D, pink dots). Synergy rates across tissues in this Bliss group were <25%, except for the bladder (29%) (Figure 3C, pink dot in the leftmost column). Half (11/21) of the tissues for this combination did not show any IDA responses. The Bliss responses of MK-1775 + cisplatin (Figure S1B) were efficacious, illustrating that efficacy extends beyond synergistic responses.

The *IDA group* had a median Bliss rate of 23% and median synergy rates of 1%. The IDA rate was highest for trametinib + LGK974 (MAP2K1; MAP2K2+PORCN), with half of the tissue types having an IDA rate exceeding 75%. The remainder showed IDA rates for this combination above 50%, with Bliss rates above 25% for the esophagus, liver, and thyroid (Figure 3C, green dots) with no instances of synergy. The median viability reduction for the IDA responses associated with this combination was 33.7% (IQR: 24.3%–48.1%).

Since cancer mutations are frequently tissue specific, we set out to investigate the association between mutation frequencies of the top 15 most frequently mutated genes (excluding TP53 mutations) across all cell lines and the top 15 synergy, Bliss, and IDA combinations. We find clustering of RAS/RAF mutations for combinations involving trametinib (mitogen-activated protein kinase [MAPK] pathway associated), but also high APC mutation rates (mostly occurring in large intestine) in IDA, confirming the intuition that gene-specific mutation rates can be associated with response class (Figure S2).

Next, we examined the relationship between class frequency and viability across combinations for each tissue-class pair. Viability was strongly correlated with frequency, with synergy being the least frequent class with the lowest viability (highest reduction in viability) and IDA being the most frequent class with the smallest reduction in viability (Figure 3D), and this also held for individual tissues, for example, lung (Figure 3E), but not for the hematopoietic and lymphoid lineage, which showed low viability across all three classes (Figure 3F).

In summary, leveraging our large panel, we classified cell lines into three response classes that convey a combination benefit: drug combinations that positively interact (synergy), act independently on a cell line (Bliss), or work through IDA. We found a positive correlation between the class frequency and viability, in which the class that was least frequent (synergy) also had the lowest viability values. This relation existed across tissues, except for the hematopoietic and lymphoid lineage. In general, combinations with higher rates of synergy also tend to show higher rates of Bliss and IDA. While being less common, combinations can show higher rates of efficacy (>50% viability reduction) within a tissue context through Bliss or IDA, rather than through synergy. However, combinations that show a higher synergy rate also show a higher Bliss and IDA-associated viability reduction. Importantly, we found that all combinations had an enhanced effect over single agents, even though the frequency of occurrence was variable.

Efficacious combination benefit

Combination response metrics such as synergy reflect the excess over Bliss. They do not capture the absolute viability effect of the combination on cells. While many studies focus on drug synergy, as we demonstrate here, a combination can be efficacious even when it is not synergistic. Hence, it can have potent cytotoxic effects and be potentially clinically effective. To capture efficacious combinations from different response classes in a single entity, we introduce the concept of ECB. ECB reflects combinations that induce at least a 50% point viability reduction, which can originate from synergy, Bliss, or IDA response classes.

The median ECB rate across all combination-tissue pairs was 29%, ranging from 14% in liver to 45% in the peripheral nervous system. We grouped ECB profiles by similarity using hierarchical clustering (1,071 combination-tissue pairs) (Figure 4A and Table S3). Several combinations had low tissue specificity, marked by similar rates across tissues. An example of this is MK-1775 + pictilisib (WEE1/PLK1, PI3K, cyan-1), which has a high ECB rate (>68%) in all tissues except endometrium. Most combinations in clinical trials across various cancers have low tissue specificity (Figures 4A, clinicaltrials.gov, April 2021; Table S4). Other combinations showed tissue specificity, but most are not in trials. For example, trametinib + olaparib (MEK1/2, PARP1/2, cyan-2) had a high ECB rate in skin cell lines (74%). Similarly, lapatinib + MK2206 (EGFR/ERBB2, AKT1/2, cyan-3) is in two tissue-specific (breast) trials (NCT01281163 and NCT01245205) and showed high ECB rates not only in breast (72%) but also in head and neck (93%), esophagus (83%), and stomach (78%). Other combinations with lapatinib showed similarly high ECB values for these tissue types, where

Figure 2. Classification of responses
(A) Example group of 11 colon and 9 lung cell lines to be classified.
(B) "Mock" drug combination for profiling.
(C) Screen for combination drug effects using an anchored setup.
(D) Obtain sensitivity parameters for each cell line in both tissue populations.
(E) In the first phase (green block), we classify on the cell line level using the decision rules in the boxes.
(F) In the final phase (pink box), we reclassify the HSA responses on the tissue population level into IDA or single-agent (SA)-driven responses.

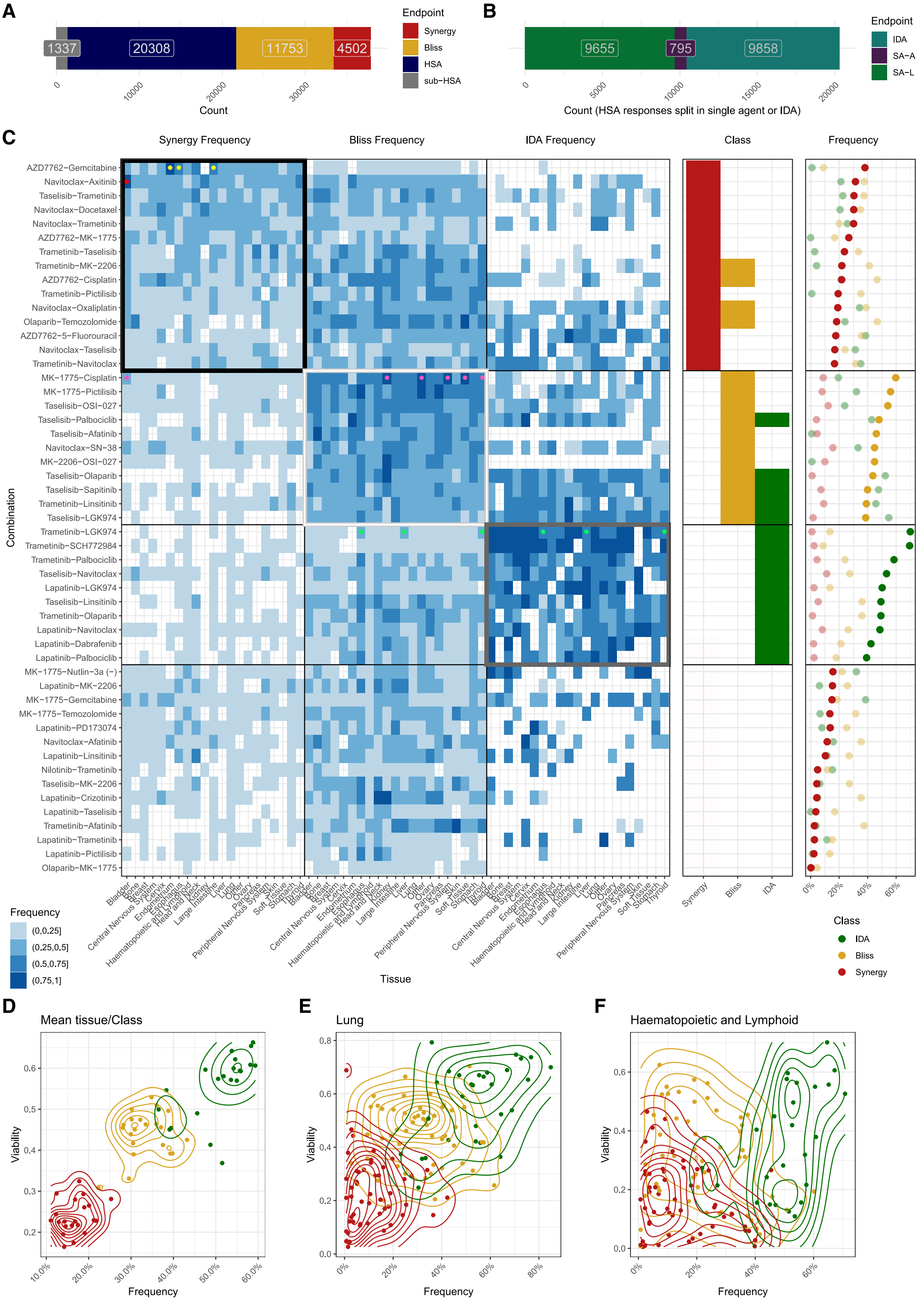

(legend on next page)

the clustering is most pronounced for esophageal and head and neck cancers.

We searched for associations between molecular features (*n* = 3,420, see STAR Methods) and ECB status to identify (sub)populations of cell lines with high ECB rates in the pan- and per-tissue populations. We found biomarkers (false discovery rate <0.05, Benjamini-Hochberg correction) for 353 combination-tissue pairs, with a median of 4 biomarkers (IQR: 1–22.75) per combination-tissue pair. For 47 combinations, we identified pan-tissue ECB biomarkers, with a median count of 225 biomarkers per combination (mean = 335, IQR: 77–496) (Table S5). We identified a median of 16 combinations with per-tissue biomarkers (IQR: 11–22) and a median of 7 tissues with at least one biomarker per combination (IQR: 5–9).

The most significant associations from the pan-tissue biomarker analysis involved combinations with an MEK (trametinib) and a PI3K (taselisib, pictilisib) or an AKT (MK2206) inhibitor (Figure S2A). The gene expression of ETV4, DUSP6, ETV5, and FOSL1 is in the top-5 biomarkers for each combination. These genes are markers of MAPK activation,[15,16] indicating that MAPK activation is associated with ECB for combinations of MEK with PI3K or AKT inhibitors. A per-tissue biomarker analysis (Figure S2B) revealed higher biomarker diversity than the pan-tissue analysis. At the same time, the number of biomarkers detected per combination, across or within all tissues, varied substantially. We observed a relationship between the number of cell lines per tissue and the number of biomarkers, with hematopoietic and lung yielding the most per-tissue biomarkers (Figure S2C).

The combination of trametinib + pictilisib yielded the strongest pan-tissue biomarker (ETV4 expression) (Figure 4B). High ETV4 expression was strongly associated with ECB status in pan-tissue and in several per-tissue analyses (Figure 4C). When high ETV4 expression (above median) is used to stratify cell lines, the ECB rate is substantially increased for pan-tissue (53%–67%) and for PNS (57%–90%) and lung (47%–66%) (Figure 4C). In a tissue-specific analysis, we found that for lung, MYEOV expression, a gene implicated in non-small cell lung cancer prognosis,[17] had the strongest association with trametinib + pictilisib ECB rates when considering only lung cell lines.

Next, we aggregated our experimental data with orthogonal evidence to identify combinations for follow-up. To this end, we included the following parameters: the tissue type, the ECB rate, information about current clinical trials for the combination, and the availability of a biomarker. For example, in the lung, trametinib + pictilisib achieves an ECB rate of 47% with both synergy and Bliss contributing to the ECB rate. The ECB rate increases to 75% when stratifying based on MYEOV expression. There are currently no trials for this combination-tissue-biomarker tuple (Figure 4E).

The biomarker results revealed that combinations with targeted (RTK) agents yielded the strongest biomarkers (Figure S3A). The combination involving a chemotherapeutic with the strongest, significant association was olaparib + temozolomide (PARP1/2, alkylating agent). We identified 594 pan-tissue ECB biomarkers (Figure S3C) for this combination, with SLFN11 expression being the strongest candidate (Figures 4F and 3G, pan-tissue). Additionally, SLFN11 expression identified subpopulations with high ECB rates within specific tissues. It increased the ECB rate from 11% to 20% in lung lines and 47% to 77% in bone (Figure 4G). In the per-tissue analysis (Figure S3B), we identified LINGO1 expression in bone as a biomarker for this combination, identifying a subpopulation with above median LINGO1 expression and an ECB rate of 88%, increasing the unstratified ECB rate by 38% (Figures 4H and 4I). High expression levels of LINGO1 have been associated with the bone cancer Ewing's sarcoma[18] (Figure S3D). The combination of olaparib + temozolomide is in a phase 2 trial for leiomyosarcoma and large intestine, suggesting an acceptable safety profile (Figure 4I).

We frequently observed that pan-tissue combination biomarkers were also tissue-specific biomarkers. The combination MK1775 + cisplatin (WEE1/PLK1, DNA crosslinking agent) was an exception in breast cell lines. In the unstratified breast (cell line) population, we found a 56% ECB rate, and a phase 2 clinical trial in triple-negative breast cancer (TNBC) is currently open for this combination (NCT03012477). Most of the ECB-positive lines were of the basal-like subtype[19] (Figure S3E), which is most similar to TNBC, matching the trial's inclusion criteria. For the pan-tissue analysis, the best biomarker (IGFBPL1) only marginally increased the ECB rate by 3% (from 56% to 59%) in the breast. In contrast, the per-tissue analysis identified TFF1 and MISP expression that increased the ECB rate to 87% and 83%, respectively (Figures 4J, 4K, and 4L). TFF1 is also known as breast cancer estrogen-inducible protein,[20] while MISP is a PLK1 substrate required for spindle orientation.[21] Thus, TFF1 points to hormone-related biology, while PLK1 is a target of MK1775. Both biomarkers were associated with the PAM50 basal subtype (Figure S3G) and are exclusive to breast (Figure 4L).

We compared the ECB classification results we obtained in this study with those from a recent screening of 2,025 clinically

Figure 3. Outcome of classification of responses

(A) Distribution of response classes (*n* = 37,900).

(B) Distribution of HSA responses after classifying HSA into SA-driven or IDA.

(C) Frequency of synergy, Bliss, and IDA responses across all 1,071 combination-tissue pairs. The 51 combinations are sorted by overall frequency of synergy at the top, then followed by sorting on the overall frequency of Bliss, and finally sorted by the overall frequency of IDA. Tissue-specific responses are displayed as three vertical blocks representing synergy, Bliss, and IDA responses, with the tissue types represented in the same (alphabetical) order in each block. Each cell represents the class frequency in the tissue (column) for a given combination (row). Empty cells represent a zero class frequency. A black, white, and gray box represent the top synergy, Bliss, and IDA combinations. Marginal percentages are provided for combinations (right). Neon-colored dots indicate the drug pair-tissue combinations mentioned in the main text.

(D–F) Scatterplots of viability against frequency for all combination benefit class-tissue pairs (D), for all combination-class pairs in the lung (E) and the hematopoietic and lymphoid lineage (F).

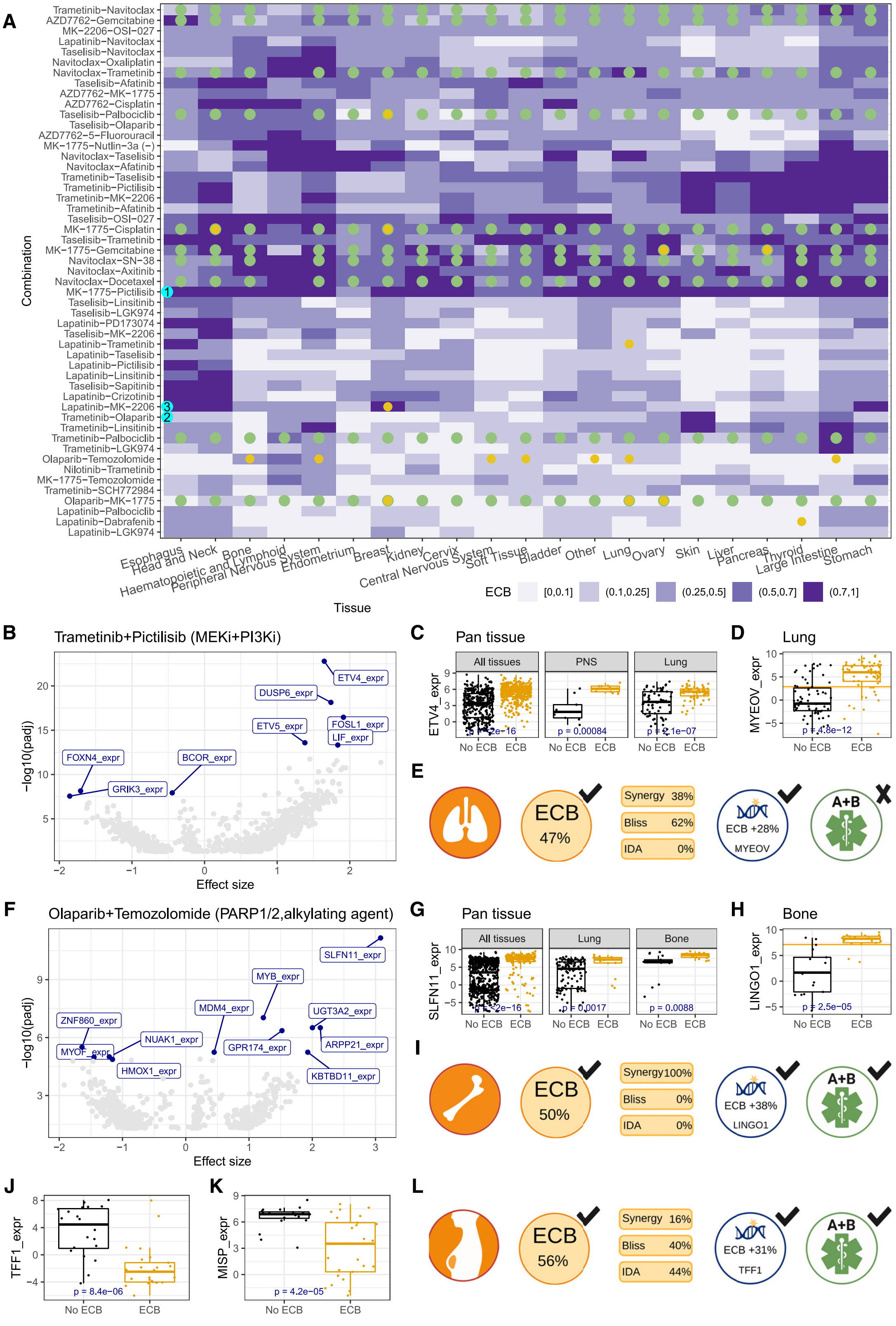

(legend on next page)

relevant drug combinations for breast, large intestine, and pancreas conducted on the same platform.[10] Of the 51 combinations, 49 overlapped, with 46 having the same anchor drug concentrations between the two datasets. The Jaaks dataset showed that 16.4% of the responses were synergistic, while our tissue-matched data showed a rate of 12.3%. We further compared the synergy frequencies between the two screens. We found good concordance (Figure S4A, Spearman r = 0.81, 0.89, and 0.9 for breast, large intestine, and pancreas, respectively). We also found similar levels of agreement with the ECB rate estimates between the two screens (Figure S4B) (Spearman r = 0.89, 0.8, and 0.9 for breast, large intestine, and pancreas, respectively). These findings indicate that both the screening platform and the analysis framework provide consistent and reliable results across independent datasets. Finally, we found that the frequency of observing ECB in a combination is associated with overall efficacy (Figure S5A, r = 0.57, p = 1.5e−5), even after removing the synergistic responses (Figure S5B, r = 0.48, p = 3.7e−4). The latter suggests that efficacious responses are not solely due to synergy but also due to Bliss and IDA responses (Figure S5C, r = 67, p = 5.2e−6).

In summary, ECB identifies combinations that elicit treatment benefits. Biomarker stratification defines the benefiting subgroup more precisely and could provide directions for further mechanistic understanding and more refined trial design.

Comparison with independent PDX combination cohort

To test the utility of ECB, we compared it with an independent drug combination study using patient-derived xenograft (PDX) models from multiple cancer types.[22] We considered the subset of combinations that had PDX response data available for the combination and both single agents and for which we could match drug targets and tissues with our cell line screening dataset. We used hazard ratios to quantify response in the PDX data, using tumor volume doubling as a survival event.[22] Fewer tumor volume doubling events means a better response and results in a lower hazard ratio.

There were nine target-matched combinations shared between the PDX and screening data. We excluded the IGF1R-MEK combination as a known false report drug interaction in the *in vitro* setting.[22] Additionally, we excluded combinations with the CDK4/6 inhibitor, LEE011, as the target-matched drug in our screen (palbociclib) appeared to affect viability only very minimally *in vitro* as compared to PDXs, in contrast to the other compounds (Figure S6B). This may be because the PDX dose was two orders of magnitude higher (150 mg/kg LEE011 equates to 345 μM at 1 kg/L) than for cell lines (4 μM, GDSC2). Most other drugs had efficacy in both the cell lines and the PDX models.

First, we considered the combination of a BRAF + EGFR inhibitor, which is an FDA-approved treatment for patients with BRAF mutant colon cancer.[23,24] Consistent with clinical activity, BRAF-mutant large intestine cell lines have an 87.5% ECB rate (7 of 8 cell lines) in response to the combination of dabrafenib + lapatinib (Figure 5A). In contrast, the ECB rate was only 36% (14 of the 39 large intestine lines) for unstratified lines. BRAF-mutant PDX tumors (Figure 5B) responded to the combination, but the effect was not statistically significant (p = 0.33) due to the small number (n = 6) of BRAF-mutant models in the PDX study.

Of all combinations shared between the cell line screen and the PDX data, a combination targeting PI3K + MEK showed the best match. More specifically, BYL719 + binimetinib showed a 100% ECB rate in large intestine in our cell line screen (and 52% ECB for taselisib + pictilisib), and the target-matched combination of trametinib + pictilisib showed the lowest hazard ratio in the PDX screen. Specifically, when comparing the combination effect in the PDXs against the effect of the single treatments (BYL719 and binimetinib) and the untreated cohort, the combination showed highly significant hazard ratios of 0.28, 0.18, and 0.02 with associated p values of 3.9e−5, 1.7e−6, and 4.8e−5, respectively (Figure 5C).

Next, we investigated for shared combinations (N = 7) whether we could detect an overall trend between the fraction of synergistic cell lines and the hazard ratio of a combination against the placebo. Across combinations, the synergy rate obtained in cell lines is not associated with the hazard ratio achieved in the PDX models (p = 0.19) (Figure 5D). However, across combinations, the ECB rate in cell lines correlates significantly with the hazard ratio in PDX models (p = 0.0011) (Figure 5E). This

Figure 4. Biomarkers for ECB status across and within tissues

(A) Clustering of ECB rate by combination and tissue. Dots represent pan-tissue (green) or tissue-specific (yellow) clinical trials.

(B) Volcano plot showing the association between all tested omics markers and ECB for trametinib + pictilisib across all tissue types.

(C) Boxplots showing the association between ECB and ETV4 expression across all tissues (left) and for the peripheral nervous system (PNS) (middle) and lung (right).

(D) Boxplot for the tissue-specific biomarker, MYEOV expression, for trametinib + pictilisib in the lung, the horizontal yellow line represents the median MYEOV expression.

(E) Graphical summary for trametinib + pictilisib in lung shows the tissue-specific ECB rate with the contribution of the response classes to the ECB rate, availability and identity of biomarkers (MYEOV expression) and associated ECB rate improvement (threshold: median expression), and trial availability.

(F) The volcano plot shows the association between all tested omics markers and ECB for olaparib + temozolomide across all tissue types.

(G) Boxplots of the association between ECB and SLFN11 expression across all tissues (left) and for lung (middle) and bone (right).

(H) Boxplot for the bone-specific biomarker, LINGO1 expression, for olaparib + temozolomide with the yellow horizontal line representing the median LINGO1 expression.

(I) Graphical summary for olaparib + temozolomide in bone. This vignette shows (1) the tissue type where the combination is (most) effective, (2) the ECB score and the relative contributions of the different interaction classes (synergy, Bliss, and IDA) to the ECB score, (3) biomarkers for the combination, and (4) clinical trial evidence for this combination.

(J) Boxplot showing the association between ECB and TFF1 expression for MK1775 + cisplatin in breast.

(K) Boxplot showing the association between ECB and MISP expression for MK1775 + cisplatin in breast.

(L) Graphical summary of MK1775 + cisplatin in breast. All boxplots show the median and the 25% and 75% quantiles, p values are from t tests.

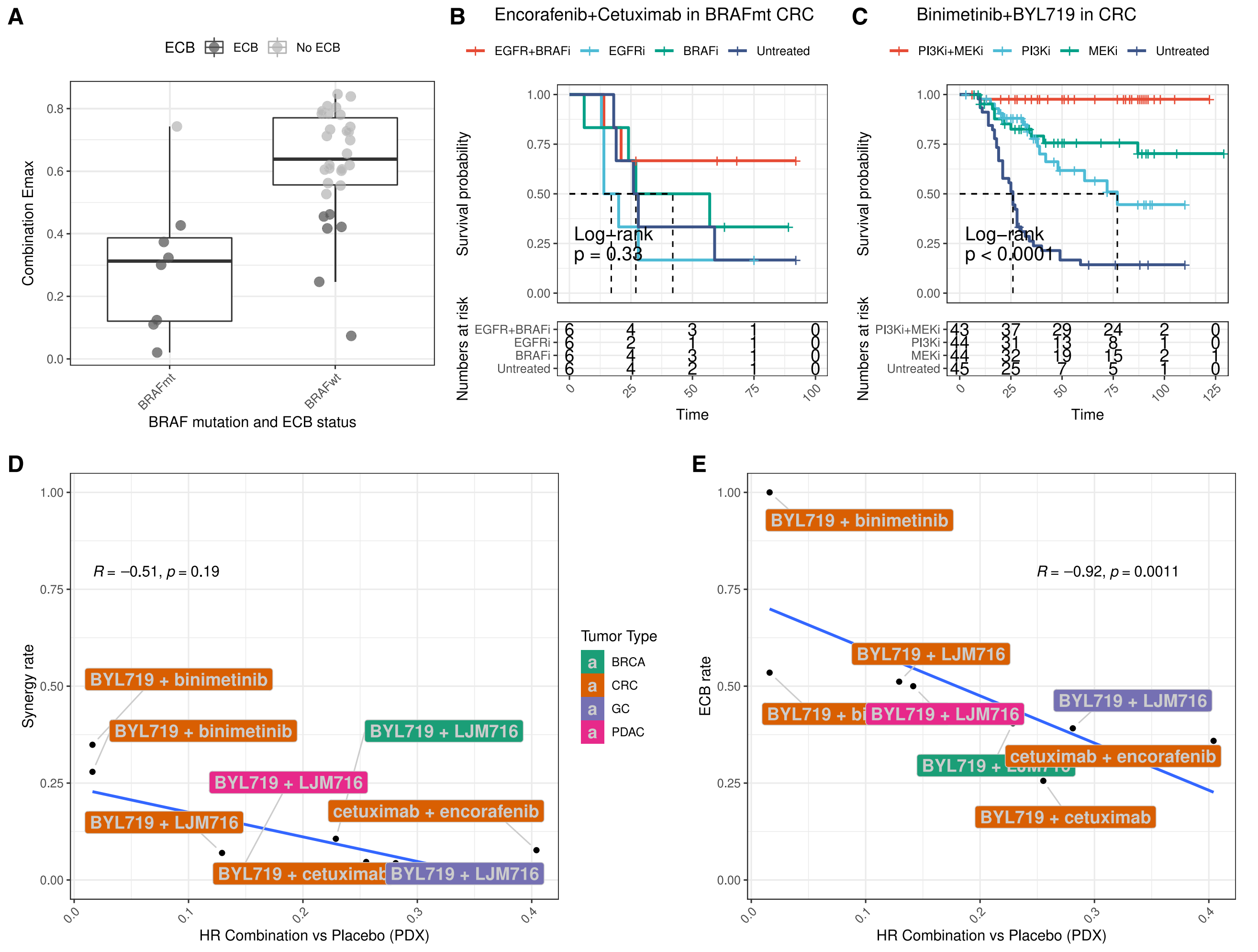

Figure 5. Comparison with PDX data

(A) Boxplots of combination Emax for cetuximab + dabrafenib (EGFR+BRAF), grouped by BRAF mutation status.

(B) Kaplan-Meier curves of BRAF-mutant colorectal PDX models treated with a combination of EGFR and BRAF inhibitors (encorafenib + cetuximab) indicated as "EGFR1+BRAFi," single agents ("EGFRi"/"BRAFi"), or vehicle ("Untreated").

(C) Kaplan-Meier curves of all colorectal PDX models treated with PI3K and MEK inhibitors (binimetinib + BYL719) indicated as "PI3Ki+MEKi," single agents ("PI3Ki"/"MEKi"), or vehicle ("Untreated").

(D) Scatterplot of the PDX hazard ratio versus cell line synergy rate for each combination. We show R and p values for Spearman correlation.

(E) Scatterplot of PDX hazard ratio versus cell line ECB rate for each combination. We show R and p values for Spearman correlation.

correlation supports the *in vivo* efficacy of combinations identified in our cell line screen. Taken together, synergy alone fails to predict combination response in PDX models, which establishes that the ECB rate, which includes synergy, Bliss, and IDA responses, best captures the *in vivo* combination response in PDX models.

DISCUSSION

Combining anticancer drugs can enhance their activity against tumor cells through synergistic or additive interactions of the constituent drugs within a single cell or can be more effective through independent action in (patient) populations.[25] Here, we introduced a classification framework that delineates these different mechanisms and incorporates combination efficacy.

We characterized combination treatment effects in individual cell lines as IDA, Bliss additivity, or synergy. Furthermore, while most preclinical studies have focused on increasing treatment potency as characterized by lower IC50 (Molar) values, in our framework, we also employ efficacious combination benefit, as represented by a minimum cell viability reduction. ECB is a measure of combination efficacy independent of the specific mode (synergy, bliss, and IDA) through which combination treatment effects are achieved. Notably, although synergy was associated with the most potent effects, many Bliss additive combinations were similarly efficacious, suggesting they could lead to meaningful clinical activity.

The many cell lines screened also enabled us to classify IDA response[11] for combinations in each tissue type. We identified combinations that target separate cell lines but increase overall

response at the cell line population level, analogous to heterogeneous patient populations. Since IDA has a role in the clinical activity of existing combination therapies,[11] and both Bliss and IDA were more common than synergy, our analysis could help identify effective drug combinations beyond those working through classical drug synergy. Collectively, our framework for classifying combination effects at the cell line and population level provides a rich resource for studying drug interactions and expands the number of opportunities to identify effective combinations.

The screen's size substantially increases statistical power,[26] allowing us to identify biomarkers of combination response. Our dataset contains at least six times as many models as most previous studies.[8,10] This revealed a rich landscape of combination treatment activity biomarkers, with all but one combination associated with a molecular marker. We combined ECB with clinical trial information (reflecting clinical precedence and tolerability in patients) and combination biomarkers (allowing patient stratification). Combining several preclinical and clinical lines of evidence makes this integrative approach useful for constraining the search space for clinically effective combinations.

The dataset and analysis framework reported is a rich resource for investigating the biological mechanisms contributing to drug combination activity. These data build on and extend the Cancer Dependency Map initiative[27]—an effort to map cancer vulnerabilities systematically—by introducing an orthogonal data type. While the anchor-library design of the screening platform was chosen to maximize throughput, hence fitting our signal-finding objective, and delivering 71% more efficiency than a full matrix design, it does have the limitation that the full range of combinations of concentrations of the pair of screened drugs is not explored. An advantage of these data is our use of existing advanced oncology drugs, which should accelerate clinical translation compared, for example, to new target discovery. For most combinations, we cannot build models with sufficient accuracy to predict efficacious combinations for specific patients. Generating additional cell line sensitivity data to cover a broader range of combinations in specific molecular backgrounds and incorporating additional molecular data types such as proteomics[28] could enhance future modeling efforts and unveil the principles of drug interactions. Additionally, incorporating dynamic single-cell transcriptional profiling could help disentangle heterogeneous responses by providing insights into the different classes of activity (IDA, Bliss, and synergy) observed with different combinations and within different cell lines to the same combination. These and other approaches are likely necessary to achieve the long-term ambition to predict, *de novo*, which combinations will be optimal for individual patients.

Limitations of the study

We used immortalized cancer cell lines to identify effective combinations. Further development of combinations requires additional studies, including independent validation using patient-derived cancer models such as organoids and PDXs. We did not explore combinatorial toxicity, which is difficult to assay for but might be predicted.[29] Additionally, new screens are required to explore the landscape of three-drug combinations. We corroborated a subset of our findings using an independent dataset from the treatment of PDX.[22] The comparison of the cell line and PDX data was challenging because of differences in experimental design and endpoints, choice of drugs to a target, the comparability of dosages, and heterogeneity in the non-overlapping set of cancer models used for each study. Nonetheless, we observed good correspondence between combination ECB in cell lines and PDX response, supporting the value of cell line screening as an initial triage stage to nominate candidate combinations. Furthermore, our finding that ECB better captures *in vivo* responses than synergy could be useful to refine the design and interpretation of *in vivo* validation studies to better capture the complex activity profile of drug combinations in heterogeneous populations.

The large-scale datasets and framework for classifying combination effects presented here provide a resource for improving the understanding of combination responses to accelerate the preclinical development of clinically effective combinations.

STAR★METHODS

Detailed methods are provided in the online version of this paper and include the following:

- KEY RESOURCES TABLE
- RESOURCE AVAILABILITY
  - ○ Lead contact
  - ○ Materials availability
  - ○ Data and code availability
- EXPERIMENTAL MODEL AND STUDY PARTICIPANT DETAILS
  - ○ Cell lines
  - ○ Mouse models
- METHOD DETAILS
  - ○ Compounds
  - ○ Screening
- QUANTIFICATION AND STATISTICAL ANALYSIS
  - ○ General
  - ○ Assay plate quality control
  - ○ Curve fitting
  - ○ Synergy calling
  - ○ Combination response classification
  - ○ ECB biomarker inference
  - ○ Clinical trial scraping

SUPPLEMENTAL INFORMATION

Supplemental information can be found online at https://doi.org/10.1016/j.xcrm.2024.101687.

ACKNOWLEDGMENTS

The research leading to these results has received funding from the Oncode Institute and the European Research Council under the European Union's Seventh Framework Programme (FP7/2007-2013)/ERC synergy grant agreement no. 319661, COMBATCANCER. This research was also funded in whole, or in part, by the Wellcome Trust Grant 206194. For the purpose of Open Access, the author has applied a CC BY public copyright license to any author accepted manuscript version arising from this submission. We acknowledge institutional funding of the NKI by the Dutch Cancer Society. The NKI RHPC facility is acknowledged for providing computational resources and infrastructure. The graphical abstract contains art sourced from SMART Servier Medical Art. Magali Michaut and Rubayte Rahman are recognized for their input in the early stages of the project.

AUTHOR CONTRIBUTIONS

M.J.G., L.F.A.W., D.J.V., P.J., S.B., and N.A. contributed to the screening design. A.B., S.B., C.H., J.H., E.L., T.M., L.R., and C.T. contributed to the data collection. D.J.V. and H.L. contributed to the data processing. S.B., H.L., P.J., N.A., and D.J.V. have contributed to data quality control. D.J.V., P.J., and N.A. contributed to the data analysis. D.J.V., P.J., N.A., E.A.C., M.J.G., and L.F.A.W. wrote the manuscript. All authors read and approved the manuscript. M.J.G. and L.F.A.W. supervised the work.

DECLARATION OF INTERESTS

L.F.A.W. has received funding from Genmab BV and Bristol Myers Squibb.

M.J.G. has received research grants from AstraZeneca, GlaxoSmithKline, Astex Pharmaceuticals, and Mosaic Therapeutics and is the Director and consultant for Mosaic Therapeutics.

Received: January 22, 2024
Revised: May 10, 2024
Accepted: July 23, 2024
Published: August 20, 2024

REFERENCES

1. Jardim, D.L., De Melo Gagliato, D., Nikanjam, M., Barkauskas, D.A., and Kurzrock, R. (2020). Efficacy and safety of anticancer drug combinations: a meta-analysis of randomized trials with a focus on immunotherapeutics and gene-targeted compounds. OncoImmunology *9*, 1710052.
2. Hu, Q., Sun, W., Wang, C., and Gu, Z. (2016). Recent advances of cocktail chemotherapy by combination drug delivery systems. Adv. Drug Deliv. Rev. *98*, 19–34.
3. Iorio, F., Knijnenburg, T.A., Vis, D.J., Bignell, G.R., Menden, M.P., Schubert, M., Aben, N., Gonçalves, E., Barthorpe, S., Lightfoot, H., et al. (2016). A Landscape of Pharmacogenomic Interactions in Cancer. Cell *166*, 740–754.
4. Ghandi, M., Huang, F.W., Jané-Valbuena, J., Kryukov, G.V., Lo, C.C., McDonald, E.R., 3rd, Barretina, J., Gelfand, E.T., Bielski, C.M., Li, H., et al. (2019). Next-generation characterization of the Cancer Cell Line Encyclopedia. Nature *569*, 503–508.
5. Barretina, J., Caponigro, G., Stransky, N., Venkatesan, K., Margolin, A.A., Kim, S., Wilson, C.J., Lehár, J., Kryukov, G.V., Sonkin, D., et al. (2012). The Cancer Cell Line Encyclopedia enables predictive modelling of anticancer drug sensitivity. Nature *483*, 603–607.
6. O'Neil, J., Benita, Y., Feldman, I., Chenard, M., Roberts, B., Liu, Y., Li, J., Kral, A., Lejnine, S., Loboda, A., et al. (2016). An Unbiased Oncology Compound Screen to Identify Novel Combination Strategies. Mol. Cancer Therapeut. *15*, 1155–1162.
7. Flobak, Å., Niederdorfer, B., Nakstad, V.T., Thommesen, L., Klinkenberg, G., and Lægreid, A. (2019). A high-throughput drug combination screen of targeted small molecule inhibitors in cancer cell lines. Sci. Data *6*, 237.
8. Holbeck, S.L., Camalier, R., Crowell, J.A., Govindharajulu, J.P., Hollingshead, M., Anderson, L.W., Polley, E., Rubinstein, L., Srivastava, A., Wilsker, D., et al. (2017). The National Cancer Institute ALMANAC: A Comprehensive Screening Resource for the Detection of Anticancer Drug Pairs with Enhanced Therapeutic Activity. Cancer Res. *77*, 3564–3576.
9. Seashore-Ludlow, B., Rees, M.G., Cheah, J.H., Cokol, M., Price, E.V., Coletti, M.E., Jones, V., Bodycombe, N.E., Soule, C.K., Gould, J., et al. (2015). Harnessing Connectivity in a Large-Scale Small-Molecule Sensitivity Dataset. Cancer Discov. *5*, 1210–1223.
10. Jaaks, P., Coker, E.A., Vis, D.J., Edwards, O., Carpenter, E.F., Leto, S.M., Dwane, L., Sassi, F., Lightfoot, H., Barthorpe, S., et al. (2022). Effective drug combinations in breast, colon and pancreatic cancer cells. Nature *603*, 166–173.
11. Palmer, A.C., and Sorger, P.K. (2017). Combination Cancer Therapy Can Confer Benefit via Patient-to-Patient Variability without Drug Additivity or Synergy. Cell *171*, 1678–1691.e13.
12. Ling, A., and Huang, R.S. (2020). Computationally predicting clinical drug combination efficacy with cancer cell line screens and independent drug action. Nat. Commun. *11*, 5848.
13. Vis, D.J., Bombardelli, L., Lightfoot, H., Iorio, F., Garnett, M.J., and Wessels, L.F. (2016). Multilevel models improve precision and speed of IC50 estimates. Pharmacogenomics *17*, 691–700.
14. Bliss, C.I. (1939). The toxicity of poisons applied jointly1. Ann. Appl. Biol. *26*, 585–615.
15. Dry, J.R., Pavey, S., Pratilas, C.A., Harbron, C., Runswick, S., Hodgson, D., Chresta, C., McCormack, R., Byrne, N., Cockerill, M., et al. (2010). Transcriptional pathway signatures predict MEK addiction and response to selumetinib (AZD6244). Cancer Res. *70*, 2264–2273.
16. Vallejo, A., Perurena, N., Guruceaga, E., Mazur, P.K., Martinez-Canarias, S., Zandueta, C., Valencia, K., Arricibita, A., Gwinn, D., Sayles, L.C., et al. (2017). An integrative approach unveils FOSL1 as an oncogene vulnerability in KRAS-driven lung and pancreatic cancer. Nat. Commun. *8*, 14294. https://doi.org/10.1038/ncomms14294.
17. Zhang, R., and Ma, A. (2021). High expression of MYEOV reflects poor prognosis in non-small cell lung cancer. Gene *770*, 145337.
18. Morales, E., Olson, M., Iglesias, F., Dahiya, S., Luetkens, T., and Atanackovic, D. (2020). Role of immunotherapy in Ewing sarcoma. J. Immunother. Cancer *8*, e000653.
19. Parker, J.S., Mullins, M., Cheang, M.C.U., Leung, S., Voduc, D., Vickery, T., Davies, S., Fauron, C., He, X., Hu, Z., et al. (2009). Supervised risk predictor of breast cancer based on intrinsic subtypes. J. Clin. Oncol. *27*, 1160–1167.
20. Prud'homme, J.F., Fridlansky, F., Le Cunff, M., Atger, M., Mercier-Bodart, C., Pichon, M.F., and Milgrom, E. (1985). Cloning of a gene expressed in human breast cancer and regulated by estrogen in MCF-7 cells. DNA *4*, 11–21.
21. Zhu, M., Settele, F., Kotak, S., Sanchez-Pulido, L., Ehret, L., Ponting, C.P., Gönczy, P., and Hoffmann, I. (2013). MISP is a novel Plk1 substrate required for proper spindle orientation and mitotic progression. J. Cell Biol. *200*, 773–787.
22. Gao, H., Korn, J.M., Ferretti, S., Monahan, J.E., Wang, Y., Singh, M., Zhang, C., Schnell, C., Yang, G., Zhang, Y., et al. (2015). High-throughput screening using patient-derived tumor xenografts to predict clinical trial drug response. Nat. Med. *21*, 1318–1325.
23. Prahallad, A., Sun, C., Huang, S., Di Nicolantonio, F., Salazar, R., Zecchin, D., Beijersbergen, R.L., Bardelli, A., and Bernards, R. (2012). Unresponsiveness of colon cancer to BRAF(V600E) inhibition through feedback activation of EGFR. Nature *483*, 100–103.
24. Tabernero, J., Grothey, A., Van Cutsem, E., Yaeger, R., Wasan, H., Yoshino, T., Desai, J., Ciardiello, F., Loupakis, F., Hong, Y.S., et al. (2021). Encorafenib Plus Cetuximab as a New Standard of Care for Previously Treated BRAF V600E-Mutant Metastatic Colorectal Cancer: Updated Survival Results and Subgroup Analyses from the BEACON Study. J. Clin. Oncol. *39*, 273–284.
25. Lopez, J.S., and Banerji, U. (2017). Combine and conquer: challenges for targeted therapy combinations in early phase trials. Nat. Rev. Clin. Oncol. *14*, 57–66.
26. Button, K.S., Ioannidis, J.P.A., Mokrysz, C., Nosek, B.A., Flint, J., Robinson, E.S.J., and Munafò, M.R. (2013). Power failure: why small sample size undermines the reliability of neuroscience. Nat. Rev. Neurosci. *14*, 365–376.
27. Boehm, J.S., Garnett, M.J., Adams, D.J., Francies, H.E., Golub, T.R., Hahn, W.C., Iorio, F., McFarland, J.M., Parts, L., and Vazquez, F. (2021). Cancer research needs a better map. Nature *589*, 514–516.
28. Gonçalves, E., Poulos, R.C., Cai, Z., Barthorpe, S., Manda, S.S., Lucas, N., Beck, A., Bucio-Noble, D., Dausmann, M., Hall, C., and

CellPress
OPEN ACCESS

Hecker, M. (2022). Pan-cancer proteomic map of 949 human cell lines. Cancer Cell *40*, 835–849. https://doi.org/10.1016/j.ccell.2022.06.010.

29. Ianevski, A., Timonen, S., Kononov, A., Aittokallio, T., and Giri, A.K. (2020). SynToxProfiler: An interactive analysis of drug combination synergy, toxicity and efficacy. PLoS Comput. Biol. *16*, e1007604.

30. van der Meer, D., Barthorpe, S., Yang, W., Lightfoot, H., Hall, C., Gilbert, J., Francies, H.E., and Garnett, M.J. (2019). Cell Model Passports—a hub for clinical, genetic and functional datasets of preclinical cancer models. Nucleic Acids Res. *47*, D923–D929.

31. Bates, D., Mächler, M., Bolker, B., and Walker, S. (2015). Fitting linear mixed-effects models Using lme4. J. Stat. Software *67*, 1–48.

STAR★METHODS

KEY RESOURCES TABLE

REAGENT or RESOURCE	SOURCE	IDENTIFIER
Deposited data		
Cancer Facts and Figures 2021	American Cancer Society (ACS), Atlanta, Georgia, 2021	www.seer.cancer.gov/csr/1975_2018/ download_csr_datafile.php/sect_01_Table 01 csv
Patient-derived xenograft encyclopedia (PDXE)	Nature Medicine	https://doi.org/10.1038/nm.3954
Cell Model Passport details	Cell Model Passports	https://cellmodelpassports.sanger.ac.uk/passports
Classified combination response data	This publication	Tables S2 and S3
Experimental models: Cell lines		
Cell lines, as defined in Table S1	See Table S1A column AT	Table S1A
Software and algorithms		
Rocker verse (4.2)	Rocker Project	https://hub.docker.com/r/rocker/verse
Singularity (3.6.4)	Sylabs Inc.	https://github.com/sylabs/singularity/releases/
Custom code	This publication	Data S2

RESOURCE AVAILABILITY

Lead contact

Further information and requests for resources and codes should be directed to and will be fulfilled by the lead contact, Dr. L. Wessels (l.wessels@nki.nl).

Materials availability

This study did not generate any new unique reagents or models.

Data and code availability

Data and code generated for results are supplied as supplemental data with this publication. Any additional information required to reanalyze the data reported in this work paper is available from the lead contact upon request.

EXPERIMENTAL MODEL AND STUDY PARTICIPANT DETAILS

Cell lines

We acquired all cell lines from commercial cell banks. All cell lines were grown in RPMI (supplemented with 10% FBS, 1% Penicillin/Streptomycin, 1% Glucose, 1.0 mM Sodium Pyruvate) or DMEM/F12 media (supplemented with 10% FBS, 1% Penicillin/Streptomycin) at 37°C in a humidified atmosphere at 5% CO_2. We profiled all lines using a panel of 95 SNPs (Fluidigm, 96.96 Dynamic Array IFC). We also performed a short tandem repeat (STR) analysis and matched these cell line profiles to the cell line repository. More details can be found at the cell model passport website.[30]

For the biomarker analyses, we placed tissue types with fewer than ten models in a category called Other (n = 19), which contains: adrenal gland (n = 1), vulva (n = 1), testis (n = 2), uterus (n = 3), biliary tract (n = 5), and prostate (n = 7).

Mouse models

The patient-derived xenograft encyclopedia (PDXE) data[22] was used to provide a reference point from an *in vivo* model system. The hazard ratios are considered for those combinations with target-gene matches and single-agent data available for both drugs. Following the PDXE authors, we used tumor volume doubling as a proxy for an event.

METHOD DETAILS

Compounds

Compounds were sourced from commercial vendors. DMSO-solubilized compounds were stored at room temperature in low humidity (<12%), low oxygen (<2.5%) environment using Storage Pods (Roylan Developments). A fridge maintained the water-solubilized compounds at 4°C during storage. Anchor and library concentrations were drug-specific and typically did not exceed 10 μM, the

exceptions are temozolomide (30 μM) and 5-FU (20 μM). We screened the anchor drugs at two fixed concentrations with a 2, 4, or 10-fold difference between them. For the library drugs we used a seven-point 1,000-fold concentration range. The steps between each point were equidistant and represented a 3.16-fold (half-log series) concentration difference.

Screening

Cells were transferred into 1536-well plates in 7.5 μL of their respective growth medium using XRD384 (FluidX) dispensers. An optimized seeding density ensured that each cell line was in the exponential growth phase at the end of the assay. The maximum density tested varied based on cell type, typically 5,000 cells/well for suspension cells and 1,250 cells/well for adherent cells. Assay plates were incubated at 37°C in a humidified atmosphere at 5% CO_2 for 24 h and then dosed with the test compounds using an Echo555 (Labcyte). The final DMSO concentration was typically 0.2%. After dosing, plates were incubated, and the drug treatment was 72 h. Cell viability was quantified by adding 2.5 μL of CellTiter-Glo 2.0 (Promega) to each well and set at room temperature for 10 min. Luminescence was quantified using a Paradigm (Molecular Devices) plate reader.

Before the screening, seeding density optimization for all cell lines was carried out by preparing a serial dilution of cells across six seeding densities with a 2-fold dilution step. Each suspension was dispensed into 224 wells of a single 1536-well assay plate using an XRD384 (FluidX) dispenser and then incubated at 37°C in a humidified atmosphere at 5% CO_2 for 96 h. Cell viability was quantified using CellTiter-Glo 2.0 (Promega). Optimal densities selected were required to be within the assay's linear range.

QUANTIFICATION AND STATISTICAL ANALYSIS

General

The analyses were performed using R (R-project) from the rocker/verse 4.2 image (see key resources table). The singularity container definition file (supplemental data) defines additional tools and software added to the container image.

Assay plate quality control

All screening plates contained negative control wells (untreated wells, $n = 6$; DMSO-treated wells, $n = 126$) and positive control wells (blanks, or medium-only wells, $n = 28$; Staurosporine treated wells, $n = 20$; and MG-132 treated wells, $n = 20$) distributed across the plate. We used these positive and negative control wells to test whether the plates met defined quality control criteria. We applied a coefficient of variation (CV) threshold of 0.18 to the DMSO-treated negative controls (CV = σN/μN, with σN the standard deviation of the negative control and μN the mean of the negative control). Using the DMSO-treated negative control (NC1) and the two positive controls (PC1, PC2), we determined Z-factors (1–3*(σP + σN)/(|μP - μN|), with σN and σP the standard deviation of the negative and positive controls, and μN and μP the mean of the negative and positive controls, respectively). For cell lines sensitive to either positive control (NC:PC ratio >4), we compute the Z-factors. For insensitive cell lines, we computed the Z-factors using the NC:blank ratio instead. A QC threshold of 0.3 for plates was applied to the Z-factors. A cell line was sensitive to both positive controls; it had to pass Z-factor thresholds for both positive controls. We have excluded all plates that did not meet these requirements.

Curve fitting

The raw fluorescent intensity (FI) values were normalized to a relative viability scale (ranging from 0 to 1) using the positive control (PC) and negative control (NC) values. For the positive control, blank wells without any cells of drugs ($n = 28$) were used. For the negative control, both wells seeded with cells with medium only ($n = 6$) and wells seeded with cells with medium and DMSO ($n = 126$) were used. The following equation shows this normalization:

$$viability = \frac{FI - PC}{NC - PC}$$

in which FI represents the luminescent intensity of a treated cell line. The viability data is derived from seven-point dose-response assays. The seven concentrations were first mapped to a relative scale (x). Each unit difference translates to a 2-fold concentration difference, and 9 represents the maximum test concentration. All library drug dose-response curves were fitted as a 2-parameter sigmoid function:

$$f(pos_{ij}, shape_i, xmid) = \frac{1}{1+e^{-\frac{xmid - pos_{ij}}{shape_i}}}$$

The parameters inference was performed using a hierarchical mixed model.[13,31] In this model, the following effects exist; a cell line effect (i), a library drug effect (j), and a replicate effect (k). Following standard nomenclature β refers to fixed effects, and b refers to random effects.

$$pos_{ijk} = \beta_1 + b1_i + b1_{ij} + b1_{ijk}$$

$$shape_i = \beta_2 + b2_i$$

On the cell line level, both pos and shape are estimated. The slope at the inflection point is assumed to be constant for a cell line, previous work found that the slope varied most across cell lines. Since this also allows a better estimate of the cell line's slope, the estimates are improved by borrowing information from responses to other compounds for that cell line. Replicate observations of cell line/compounds were treated as individual observations and not averaged before fitting. The model used is an adapted version of the IC50 model used before[3,13] that accommodates the replicates. Furthermore, the combination dose-response fitting differs from single-agent fitting: 1) the cell line parameters ($b1_i$ and $b2_i$) were obtained from the library drug fits, and 2) f() was scaled to go from 0 up to the anchor viability (rather than from 0 to 1). To assess the quality of the fits, we computed the root-mean-square error (RMSE) between the curve fit and the observed viability. Curves with an RMSE >0.2 were excluded from the analysis.

We determined IC50 and Emax for each dose-response curve using the fitted models. The Emax values are established using the fitted parameters at the highest test concentration (x = 9). For the library curves, we call these parameters library_IC50 and library_emax. For the combination curves, we call these parameters combi_IC50 and combi_emax.

Synergy calling

The expected combination response follows the Bliss independence model[14] model, assuming that the drugs do not interact. Conceptually, every expected viability at a given library concentration on the Bliss dose-response curve is defined as the product between the anchor viability and the viability at the corresponding library concentration on the library dose-response curve. Hence, the Bliss_IC50 concentration is the same as the library_IC50 concentration, but with lower viability. The Bliss-Emax equals the product of the anchor viability and the Library-Emax, which is the viability at the highest screened library concentration.

We used ΔPotency and ΔEmax to call synergy, defined as the difference over the Bliss expectation for the combination response. We specified the model to estimate all parameters simultaneously and used the variance estimate for the library replicates to define the statistical difference between observation and expectation for potency shifts. The replicate standard deviation was $\sigma = 0.825$, translating to a 99% CI ΔPotency threshold of (0.825*2.58) 2.13 (log2 scale), or a 4.36 fold-change in terms of concentration of the inflection point. For a more straightforward interpretation, we have rounded it to the next integer value and use a ΔPotency threshold of 3 instead, representing an 8-fold difference in the concentration. In parallel, we have adopted a 20% ΔEfficacy threshold to account for a meaningful efficacy gain over Bliss independence. We classify a response as synergistic if either threshold is reached. To prevent overreliance on extrapolated dose-response curves, we only considered those responses with a Bliss_IC50 up to the maximal test concentration 2-fold.

Combination response classification

When we classify the responses into synergy, Bliss, HSA, or sub-HSA, we recognize that measurements come with uncertainty and biological variability. The screen contained repeat experiments (median is 1, IQR is 1–2, maximum is 62. We have attributed the variance of repeat experiments of the highest test concentration for drug/cell lines responses (SA library only). The estimated standard deviation was $\sigma = 0.03326$, which brings the 95%CI to 0.03326*1.96 = 0.07, or 7% on the relative viability scale. Hence, we used this 7% window around our base-class HSA. Bliss and HSA are very close in many cases, and when the difference is less than this 7% viability, we stick to the HSA label.

Next, to obtain an overview of drug combination response classes across the 37,900 combination-cell line pairs in our screen, we classified all 'anchor concentration - library - cell line' pairs and selected the highest-ranking category from the two anchor concentrations. The ranking of categories used is (high to low) synergy, Bliss, HSA, sub-HSA. In the case of a tie, the anchor concentration with the highest ΔPotency is selected.

As a final step, we re-evaluate all HSA responses by tissue type and combination. We assess by tissue whether the anchor or the library is driving the HSA group in more than 90% of the cell lines. When that is the case, we relabel those cell lines as single-agent (SA) driven. Otherwise, we relabel them as independent drug action (IDA).

ECB biomarker inference

The ECB biomarkers associations were performed using the binary ECB labels. The features explored for biomarkers were the 2500 genes with the most variable expression across the cell lines, the CiViC genes, and the genomic alterations (mutation, copy number).[3]

We identified biomarkers pantissue and for each of the included tissues. Binary features (mutations, copy number regions) that were positive in fewer than four cases, or when all cases were positive, were not considered for downstream analysis. In total, 3,420 features were considered. Using the Benjamini-Hochberg procedure, a multiple testing correction was applied to each tissue-combination pair. We used the following linear mixed model with tissue as a random effect to infer pancancer biomarkers (where 1 means the intercept):

$\text{biomarker} \sim 1 + \text{ECB} + \text{tissue}_j$; for pancan models

For the tissue specific model, we used this model:

$\text{biomarker} \sim 1 + \text{ECB}$

For classification, we used the median expression level of the biomarker as a threshold without further optimization.

Clinical trial scraping

The clinical trial scraping was performed using *rvest* (tidyverse ecosystem) that queried the website clinicaltrials.gov. The resulting data were manually curated to remove false positive hits that did not match our drug combination data. The harvesting was performed in April 2021.

Query terms were: cancer and target1 and target2.

Cell Reports Medicine, Volume *5*

Supplemental information

A pan-cancer screen identifies drug combination benefit in cancer cell lines at the individual and population level

Daniel J. Vis, Patricia Jaaks, Nanne Aben, Elizabeth A. Coker, Syd Barthorpe, Alexandra Beck, Caitlin Hall, James Hall, Howard Lightfoot, Ermira Lleshi, Tatiana Mironenko, Laura Richardson, Charlotte Tolley, Mathew J. Garnett, and Lodewyk F.A. Wessels

A pan-cancer screen identifies drug combination benefit in cancer cell lines at the individual and population level

Daniel J. Vis[1#], Patricia Jaaks[2#], Nanne Aben[1#], Elizabeth A. Coker[2], Syd Barthorpe[2], Alexandra Beck[2], Caitlin Hall[2], James Hall[2], Howard Lightfoot[2], Ermira Lleshi[2], Tatiana Mironenko[2], Laura Richardson[2], Charlotte Tolley[2], Mathew J. Garnett[2,*], Lodewyk F.A. Wessels[1,4,*]

[1] Division of Molecular Carcinogenesis, The Netherlands Cancer Institute and Oncode Institute, Amsterdam 1066 CX, The Netherlands

[2] Wellcome Sanger Institute, Cambridge, UK

[3] Department of EEMCS, Delft University of Technology, Delft 2628 CD, the Netherlands

[4] Cancer Genomics Netherlands, Uppsalalaan 8, Utrecht 3584CT, the Netherlands

[#] Equal contributors

* Corresponding authors

Supplemental Figures

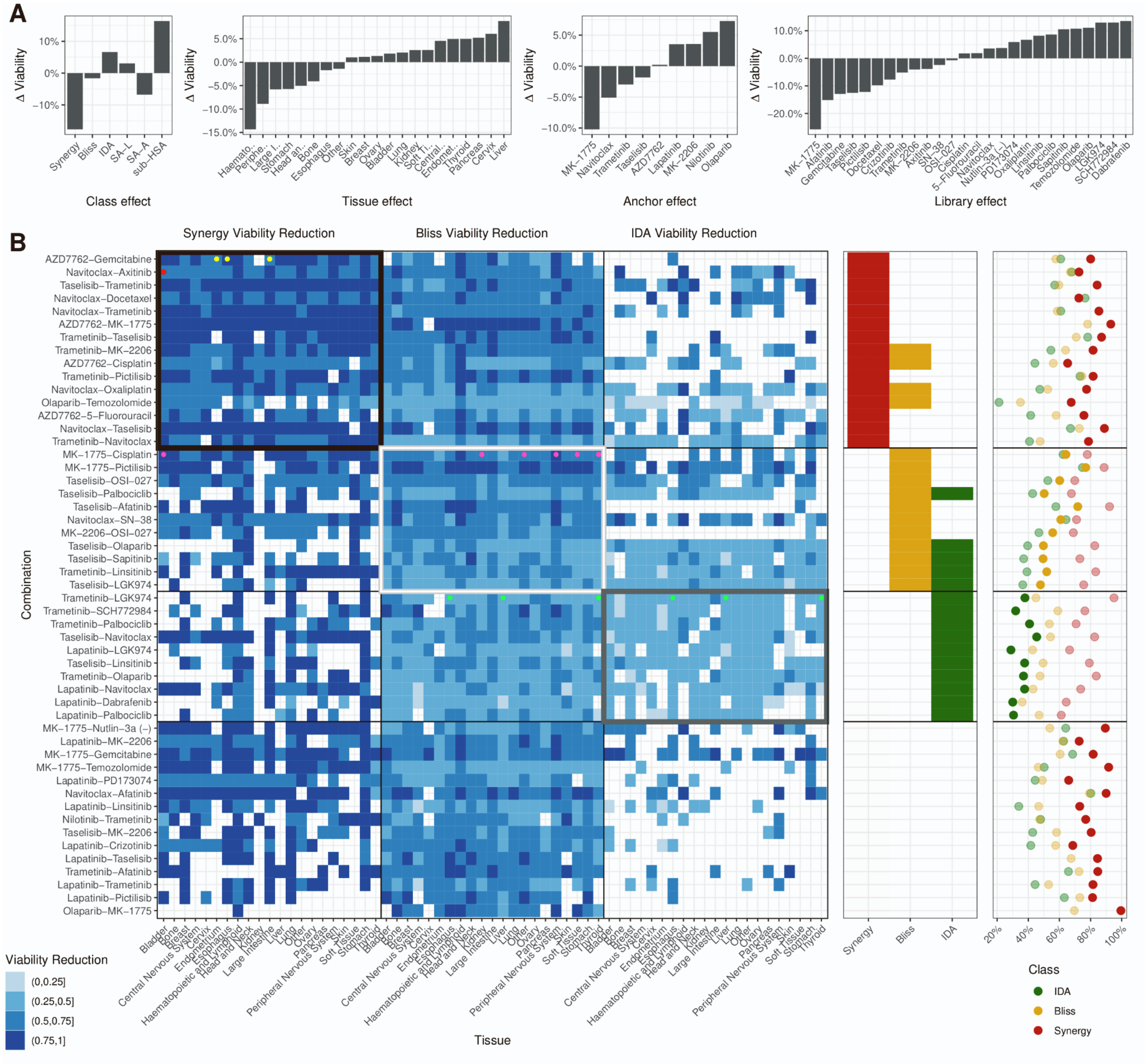

Supplemental Figure 1. **Outcome of classification of responses, related to Figure 3** (A) Decomposition of viability for individual response classes, tissue effects, anchor effects and library effects, show the general attribution of effects and their levels. (B) Mean viability reduction by tissue, combination, and combination benefit class. Empty cells mark zero class frequency. Marginal percentages are provided for combinations (right). Yellow dots indicate the drug pair-tissue combinations mentioned in the main text.

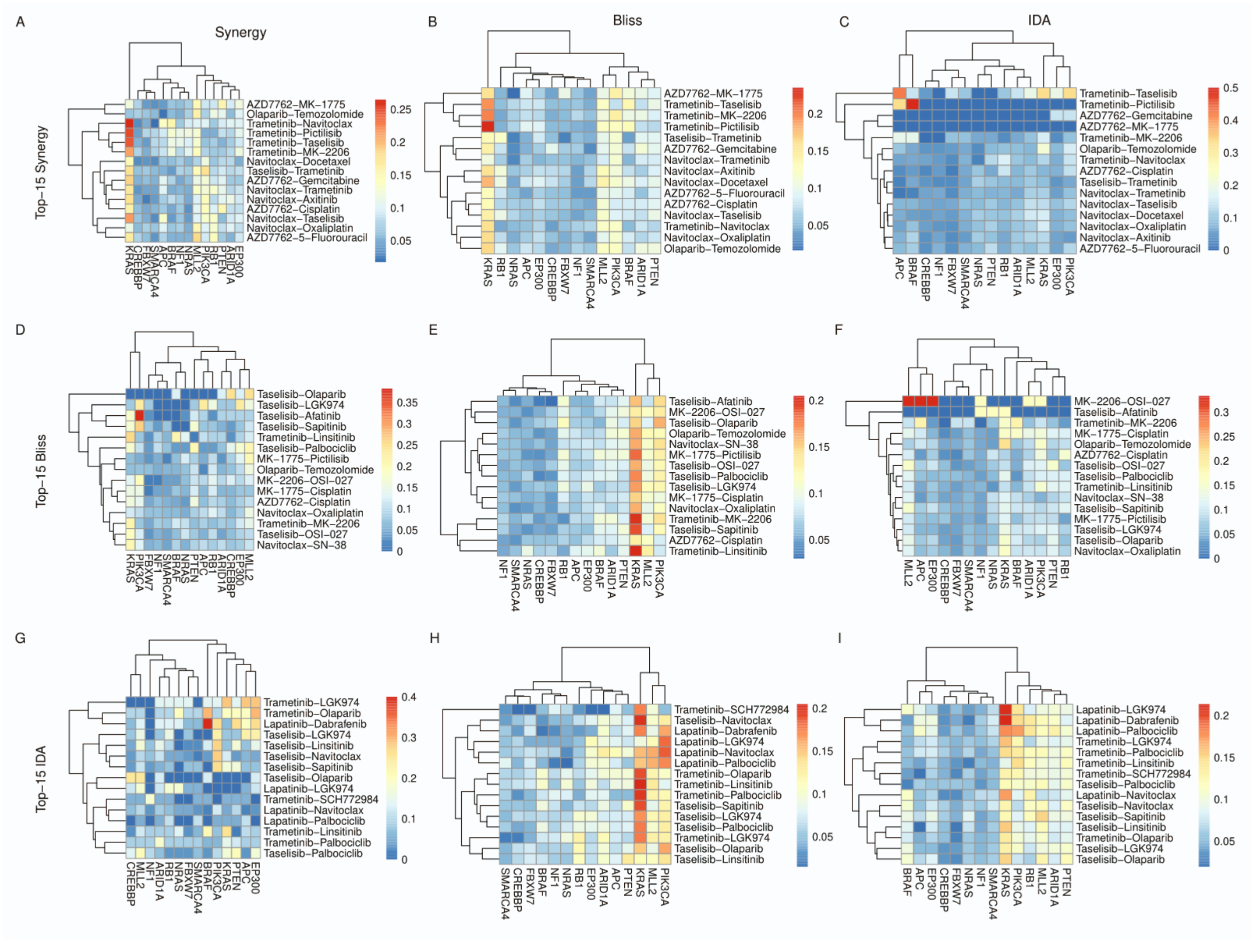

Supplemental Figure 2: **Clustered heatmaps for each of the response classes showing mutation frequencies as a function of the top 15 response classes, grouped by class, related to Figure 3**. The first row shows the top 15 synergy, the second top 15 Bliss, and the third top 15 IDA. The first column shows the mutation rates from the synergy class, the middle column from Bliss, and the third column from IDA. TP53 has been removed due to its high mutation rate to reveal more subtle patterns in the other genes. Legend indicates the mutation rate (0-1) for that class/combination.

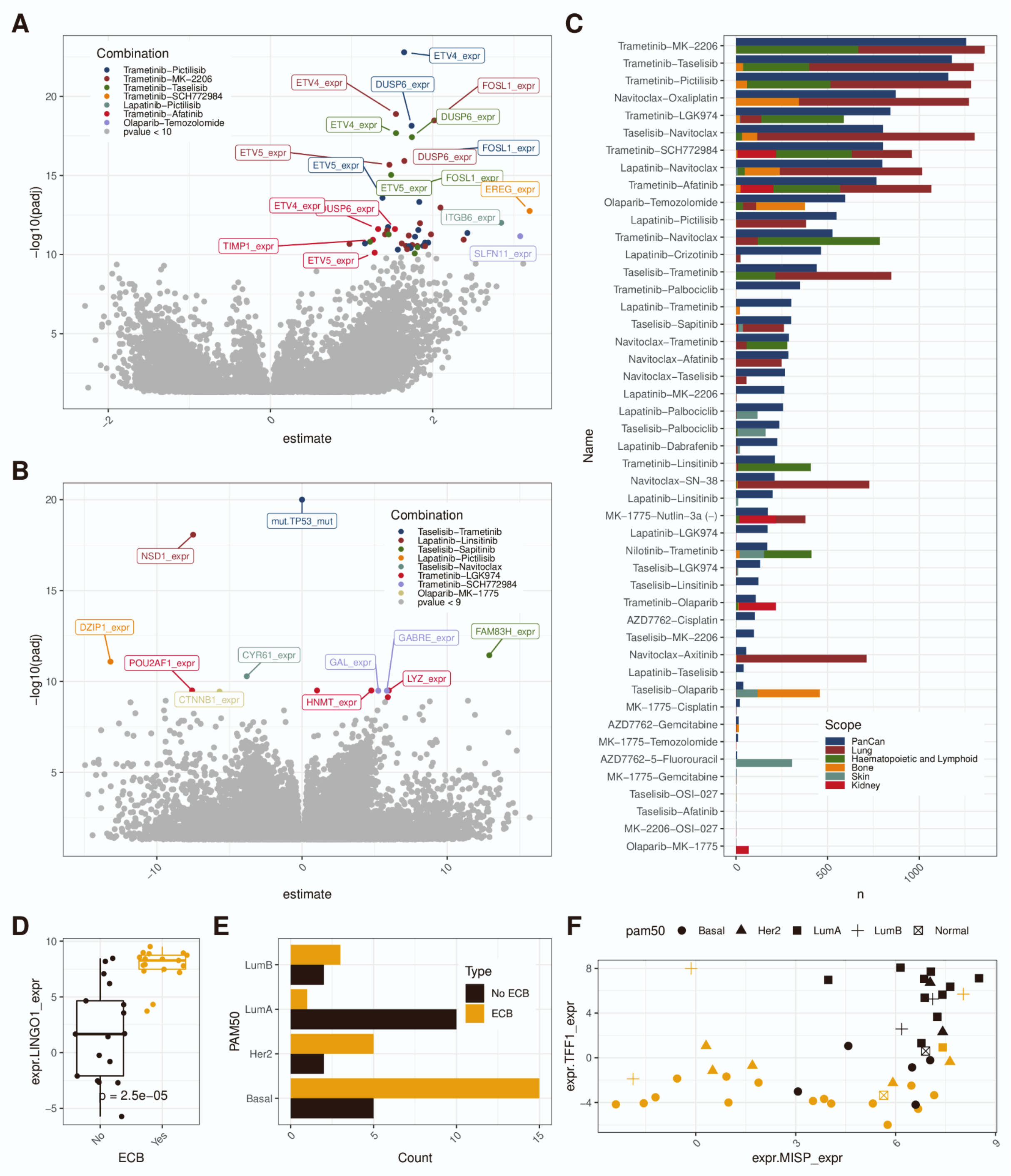

Supplemental Figure 3. Biomarkers for ECB status across and within tissues, related to Figure 4. (A) Volcano plot of pan-tissue biomarkers, showing effect size against p-value for the top five combinations per combination, labeling only the very strong associations. (B) Volcano plot of per-tissue biomarkers, -log10(p-adj) clipped at 20. (C) Count distribution of biomarkers within and across tissues. (D) Association of LINGO1 expression and ECB status in bone, boxplot shows median and 25% and 75% quantiles. (E) PAM50 subtypes and ECB status for MK1775+cisplatin

in breast. (G) Scatterplot displaying link between expression of TFF1 and MISP and PAM50 subtype.

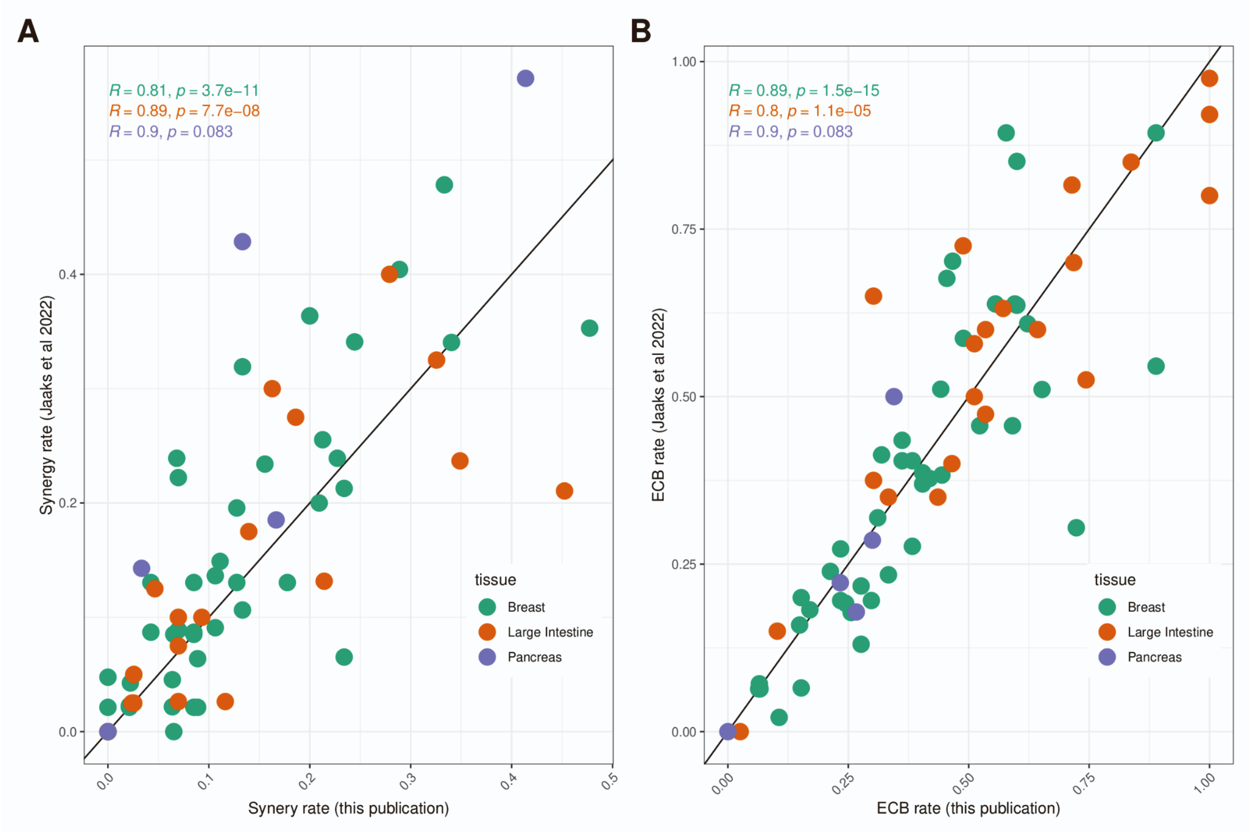

Supplemental Figure 4. Synergy and ECB rate comparison with independent screening data, related to Figure 4. (A) The synergy frequency for combinations with the same concentrations in both screens, by tissue type, and the associated per-tissue correlation. (B) The ECB frequency for the combinations with the same concentrations in both screens and their correlation by tissue type, and the associated per-tissue correlation.

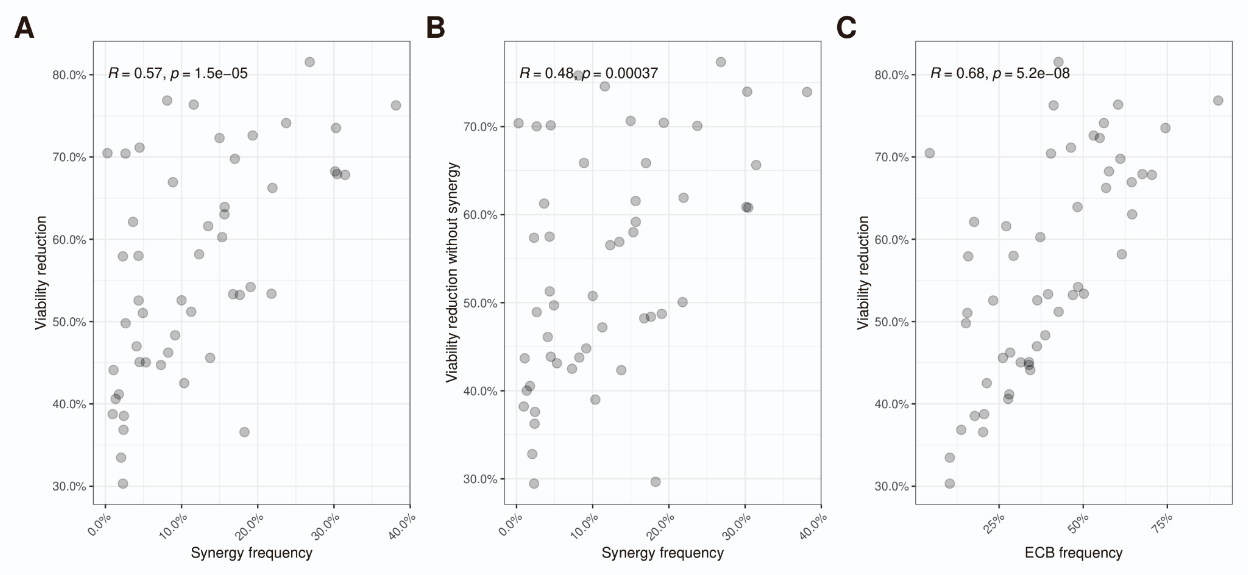

Supplemental Figure 5. Relation between synergy and efficacy, related to Figure 4. (A) Synergy count against mean viability reduction by combination. (B) Synergy count against mean viability reduction without synergy. (C) ECB count against mean viability reduction by combination.

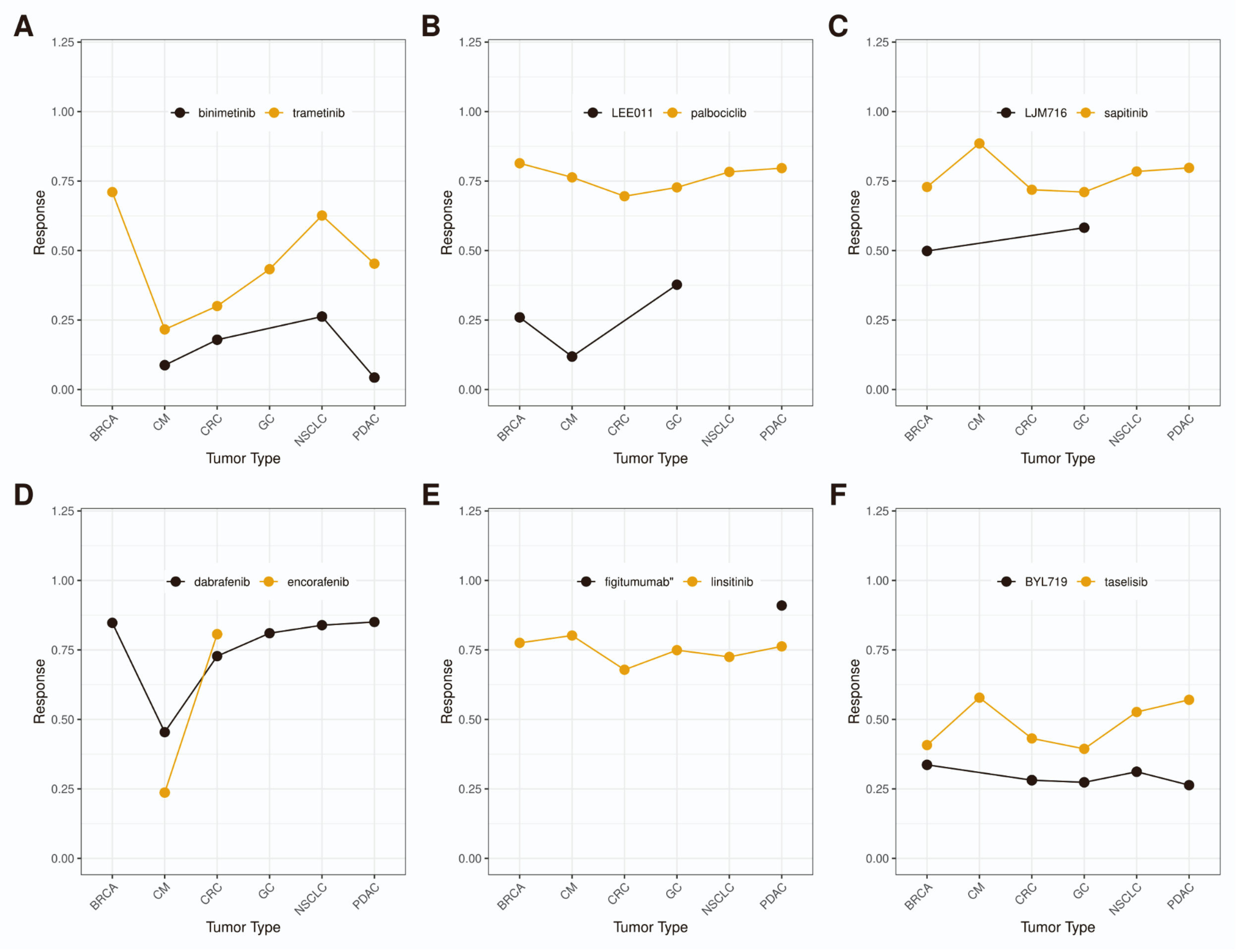

Supplemental Figure 6. Comparison of single agent effect for target-matched compounds in PDX and cell lines, related to Figure 5. (A) The hazard ratio for binimetinib (PDX, black) and mean Emax (viability at the highest concentration) for binimetinib (cell lines, yellow) show an effect of the single agent. (B) The hazard ratio for LEE011 (PDX, black) and mean Emax for palbociclib (cell lines, yellow) reveal an effect in PDX but resistance in cell lines. (C-F) Comparisons for other single-agent responses.

Supplemental Data S1: Source data and code (Data S1.zip) used for classification and figures generation, related to figures 1-5.
